# Nanostructural deformation of high-stiffness spruce wood under tension

**DOI:** 10.1038/s41598-020-79676-2

**Published:** 2021-01-11

**Authors:** Lynne H. Thomas, Clemens M. Altaner, V. Trevor Forsyth, Estelle Mossou, Craig J. Kennedy, Anne Martel, Michael C. Jarvis

**Affiliations:** 1 Department of Chemistry, University of Bath, Claverton Down, Bath, BA2 7AY UK; 2 New Zealand School of Forestry, University of Canterbury, Private Bag 4800, Christchurch, New Zealand; 3 Institut Laue-Langevin, 38042 Grenoble Cedex 9, France; 4 Partnership for Structural Biology (PSB), 38042 Grenoble Cedex 9, France; 5 Faculty of Natural Sciences, Keele University, Staffordshire, ST5 5BG UK; 6 School of Energy, Geoscience, Infrastructure and Society, Heriot Watt University, Edinburgh, EH14 4AS Scotland UK; 7 School of Chemistry, Glasgow University, Glasgow, G12 8QQ Scotland UK

**Keywords:** Biophysics, Plant sciences, Chemistry, Materials science

## Abstract

Conifer wood is an exceptionally stiff and strong material when its cellulose microfibrils are well aligned. However, it is not well understood how the polymer components cellulose, hemicelluloses and lignin co-operate to resist tensile stress in wood. From X-ray scattering, neutron scattering and spectroscopic data, collected under tension and processed by novel methods, the ordered, disordered and hemicellulose-coated cellulose components comprising each microfibril were shown to stretch together and demonstrated concerted, viscous stress relaxation facilitated by water. Different cellulose microfibrils did not all stretch to the same degree. Attempts were made to distinguish between microfibrils showing large and small elongation but these domains were shown to be similar with respect to orientation, crystalline disorder, hydration and the presence of bound xylan. These observations are consistent with a major stress transfer process between microfibrils being shear at interfaces in direct, hydrogen-bonded contact, as demonstrated by small-angle neutron scattering. If stress were transmitted between microfibrils by bridging hemicelluloses these might have been expected to show divergent stretching and relaxation behaviour, which was not observed. However lignin and hemicellulosic glucomannans may contribute to stress transfer on a larger length scale between microfibril bundles (macrofibrils).

## Introduction

Wood, the most abundant material in the biosphere, is resurging in commercial importance due to its mechanical performance, its sustainability and its potential to lock up carbon for a century or more when used in building construction^1^. Every tree contains wood with widely varying mechanical properties, exquisitely adapted to local function^2,3^. An understanding of how trees control the local properties of the wood they synthesise would not only illuminate their functional morphogenesis^2^ but would also reveal ways of growing timber for optimal commercial properties^1^. Understanding the way in which the mechanical properties of wood emerge from its nanostructure^4^ could inspire a new generation of composite materials based on nanocelluloses or on synthetic fibre polymers^4^.

New spectroscopic methods have recently advanced our knowledge of the structure of wood at the nanoscale^5,6^, although this new structural understanding has not yet been incorporated into concepts or numerical models of the mechanical performance of wood. Dry coniferous wood cell walls contain about 45–50% (by mass) of cellulose chains packed non-covalently into ~ 3 nm microfibrils. Each microfibril includes ordered domains where the *tg* C-6 conformation of the β(1,4′) linked glucose units permits a pair of axial hydrogen bonds to stabilise the covalent glycosidic linkage between the monomers^7^, leading to exceptional tensile stiffness^8^. In other domains at the microfibril surface, one of these hydrogen bonds can instead be oriented outwards to interact with other polymers or water^6,9^. The hemicellulosic polysaccharides glucomannan and xylan, making up 20–25% of the dry cell-wall mass, coil randomly when unconfined in solution but can adopt chain conformations similar to cellulose chains on the surface of microfibrils and bind to cellulose surfaces^5,10^. The aromatic polymer lignin comprises 25–30% of the dry mass and is covalently linked to hemicelluloses^11^. Cellulose microfibrils aggregate into loosely structured macrofibrils, typically 15–20 nm in diameter^12^. There are at least occasional close interactions between all the cell-wall polymers^5,13^, but much of the xylan appears to be within the macrofibrils and is bound to cellulose surfaces^5^, whereas more of the glucomannan and lignin lie between the macrofibrils^13,14^. There is little difference in chemical composition between wood with high and low tensile stiffness. Instead, the tensile properties depend predominantly on the microfibril angle, the winding angle of the cellulose in the dominant S_2_ layer of the softwood cell wall^15^.

In comparison with other non-mineralised biomaterials, wood is in general stiff and elastic^16^, but its tensile deformation has a complex mixture of elastic (reversible) plastic (irreversible) and viscous (time-dependent) components^17^. The non-elastic components are more conspicuous under the water-saturated conditions within a living tree than in dry constructional timber, and may be involved in the exceptional fracture resistance of wood, through damping external forces or internal phonons^18,19^. However we do not know much about the internal mechanisms through which wood elongates under tensile stress, nor about the contributions of its diverse polymers to these mechanisms. Simple tensile stiffness cannot be the sole adaptive consideration when the stiffest polymer component, the *tg*-conformation cellulose chains, comprise only about 20% of the dry mass^5^.

In wood with high microfibril angles, the cellulose microfibrils do not stretch appreciably but rotate towards the axial direction of the stress, and simultaneously slide along one another^2,17^. In wood with low microfibril angle, and therefore with high tensile modulus and tensile strength, much of the overall elongation comes from stretching of the cellulose chains themselves^8^ but the crystallographic strain shown by the microfibrils is less than the strain shown by the wood itself^20,21^. There must therefore be a contribution from either stretching of non-crystalline segments of the microfibrils^20^ or shear between microfibrils or macrofibrils^18,22,23^. The matrix between macrofibrils, and some of the microfibril surfaces, are hydrated^21,24,25^. Hydration reduces the tensile modulus of wood^22^.

The most detailed information on wood nanostructure has come from multidimensional NMR experiments^5^, but these cannot be conducted under controlled mechanical tension. X-ray scattering has been used to study the elongation and reorientation of cellulose under tension^20,21,26^, but not the less ordered components, although these also scatter X-rays^27^ and structural information can be derived from diffuse scattering^28^. The differential scattering from deuterium and hydrogen allows neutron diffraction experiments in which disordered, D_2_O-accessible domains can be distinguished^29^. Deuterium exchange also enables the identification of accessible polymer chains by FTIR spectroscopy^30^, where bandshifts indicate changes in the length of covalent bonds within each chain under tension^8^.

Here we describe a combination of X-ray scattering, neutron scattering and FTIR spectroscopy under tension, on Sitka spruce wood with low microfibril angle^9^. We introduce novel methods of data processing to extract small, tension-induced shifts from the diffraction and spectroscopic data. The aim was to explore the ways in which the constituent polymers of the wood cell wall interact to resist tension.

## Results

### Tensile properties

It was not possible to measure stress during the scattering and FTIR experiments on spruce wood under controlled tensile strain. Instead, the stress–strain and stress relaxation properties of the same wood samples were measured separately: see SI. Thin (< 1 mm) softwood samples differ, quantitatively at least, in their tensile properties^18^. The 20 µm thick samples used for FTIR were less stiff than the 0.5 mm samples used for the scattering experiments and showed larger irreversible and time-dependent elongation fractions (Table S1), but had quite similar stress-relaxation kinetics (Fig. S1).

### Polymer reorientation under tensile stress

Reorientation of microfibrils under tension was measured by WAXS, WANS and SANS. For all three scattering methods, with and without deuteration in the case of WANS, the orientation distribution was similar and could be modelled as a Gaussian function with standard deviation σ = 5–6° with additional, lower-intensity wings extending to about 30° from the axis (Fig. S2). It is likely that the orientation distribution extended to higher angles corresponding to microfibrils in the S1 and S3 layers of the cell wall^31^, but the complexity of the background in the diffraction patterns made such measurements impracticable. The changes in orientation distribution under tension were too small to be reliably measured by WAXS or WANS, but a slight narrowing was recorded by SANS (Fig. S3). With such a small change in orientation distribution under tension, the gain in length from rotation of the microfibrils (cosine effect)^21^ was negligible.

### Elongation of the cellulose unit cell under tension

Because each microfibril can be considered as a partially disordered crystal, the stretching of the microfibrils can be measured from changes in the position of their axial reflections in WAXS^8,20^ experiments. WANS under tension is also possible in principle but challenging due to the large sample cross-section required, and has not to our knowledge previously been attempted. The principal axial reflection in the diffraction pattern of cellulose Iβ is the 004, corresponding to a lattice plane spacing of one quarter of the unit cell, or half the length of one glucosyl unit^32^.

**Figure 1:**
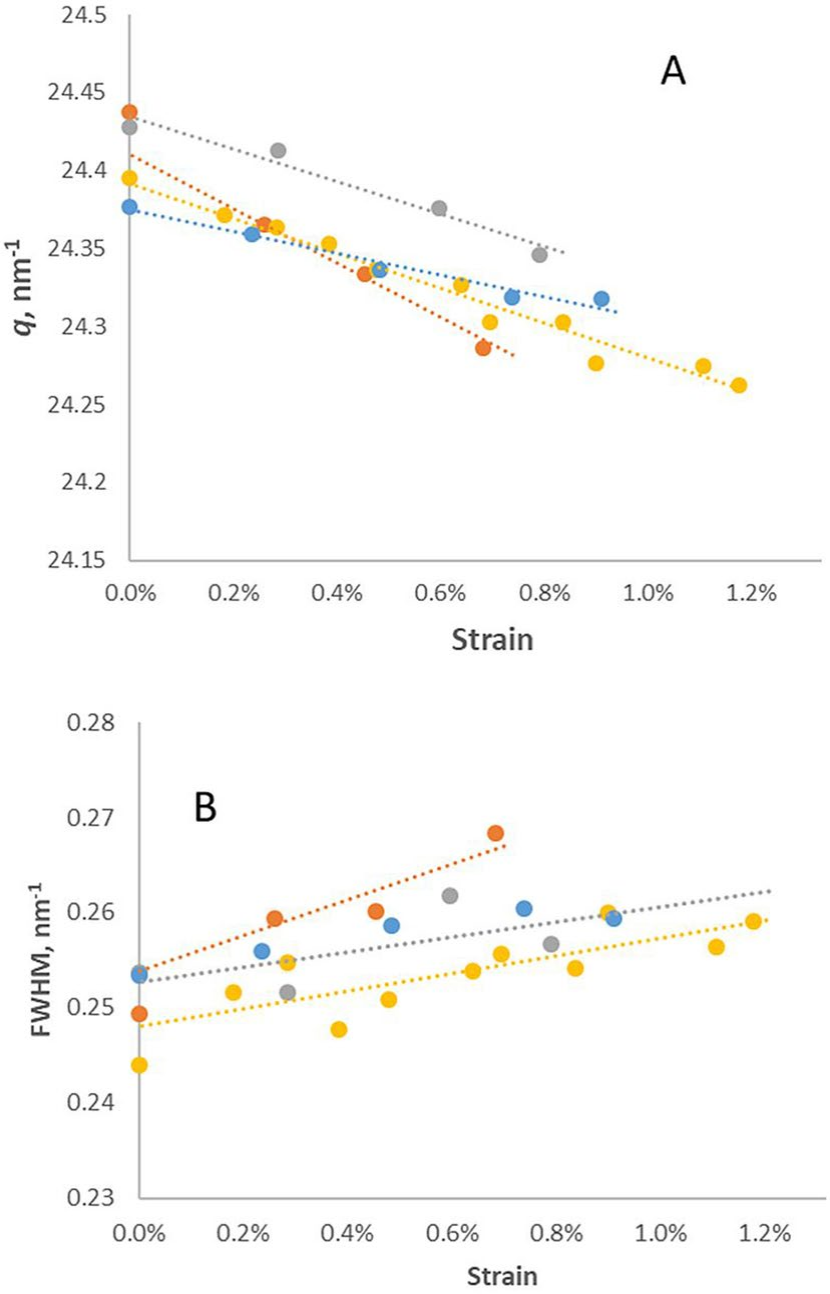
Changes in position and width of the 004 WAXS reflection, measured without tilting the sample. (**A**) Change in position: decreasing *q* with strain. Four experiments are shown separately (red, blue, green, purple), showing variation in both starting position and slope. (**B**). Strain dependence of the fitted width (FWHM) of the 004 reflection in the same four experiments. 2-Way ANOVA showed that between zero strain and 0.8% strain there were significant changes in the fitted position of the 004 reflection (*F* = 10.1, *P* < 0.05) and in fitted width (*F* = 11.2, *P* < 0.05).

When the stretching of microfibrils (crystallographic strain) was monitored directly from the 004 reflection in WAXS, it increased linearly with the macroscopic stretching of the sample up to the point of fracture (Fig. 1A). The crystallographic strain was about 25% of the macroscopic strain in the WAXS experiments. Published observations of crystallographic strain are consistently less than macroscopic strain with appropriate corrections although the ratio varies between published experiments^20,21,26^. The position of the 004 reflection with no applied strain varied significantly between samples (Fig. 1A), as has been observed previously^33,34^, perhaps due to residual growth stresses.

### Change in radial width of the 004 reflection under tension

The radial width of the 004 reflection arises partly from Scherrer broadening and partly from variation in the length of the unit cell^20^. These two contributions are not easily distinguished unless the widths of sufficient higher-order reflections can be measured^28^, which is not the case for wood. In principle variation in unit cell length across all cellulose components, together with any non-cellulosic polysaccharides that share the main-chain conformation, orientation and disaccharide unit length of cellulose, can contribute to the radial width of the 004 reflection^28^.

In all WAXS experiments where the radial width of the 004 reflection could be measured with sufficient precision, either without tilting the sample (Figs. 1B and S4) or at the appropriate tilt angle of 7° (Fig. S5), this reflection became broader as it shifted inward under tension. The broadening was quantified by using the slope integral method^8^ to estimate the change in local shift across the width of the reflection (Fig. S6). At the outer side of the reflection the shift was near zero, increasing at the inner side to almost double the shift at the centre. That is, the crystallographic strain in the microfibrils varied from near zero to about half of the macroscopic strain, with a mean ratio of about 0.25.

We looked for any features that might distinguish between microfibrils showing high and low crystallographic strain. One testable hypothesis is that microfibril segments that deviate from the mean orientation in the S2 layer of the cell wall are less stressed in tension and thus show less crystallographic strain, in the same way as all microfibrils in high-MFA wood carry less stress.

**Figure 2:**
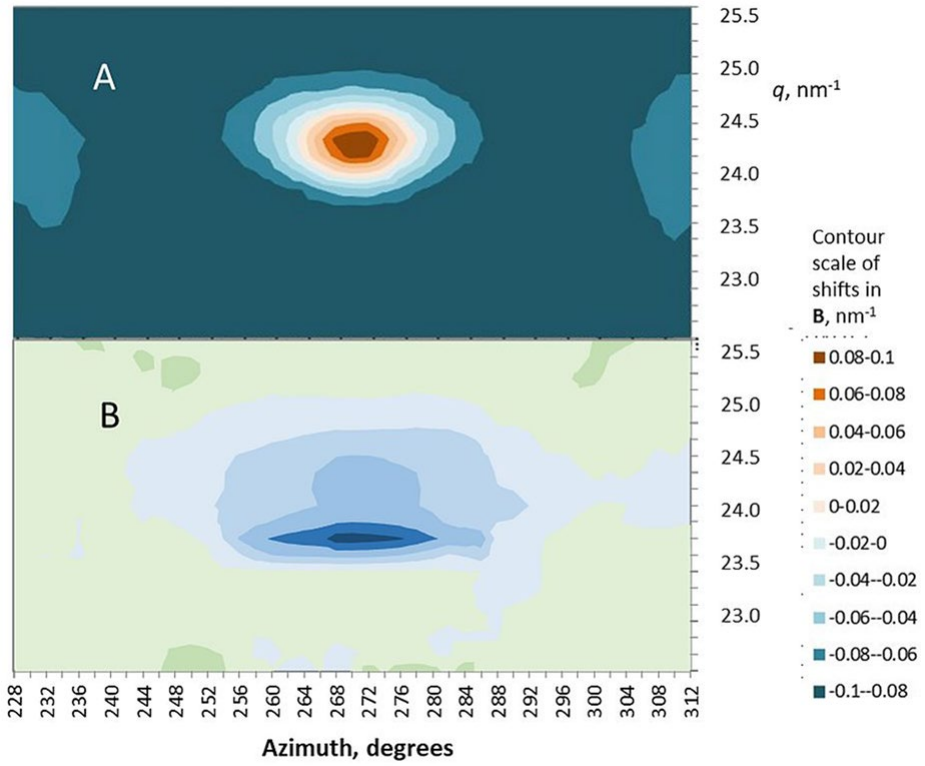
(**A**) 2D intensity map of the 004 reflection in polar co-ordinates. (**B**) local shift in *q* under 0.9% tensile strain, expressed in 2D by correlative shift mapping. Mean of four experiments. It is not practicable to indicate variation from the mean in a 2D plot but the reproducibility can be gauged from Figs. 1, S4 and S6 which are derived from 1D axial slices through the same data sets.

To examine the 004 shift in two dimensions a novel approach to the analysis of the WAXS data was developed, termed correlative shift mapping (Fig. 2). Like slope integral analysis, correlative shift mapping allows local shifts to be identified within the diffraction pattern, but the two methods are based on different mathematical principles. The largest shift was concentrated along the lower (inner) edge of the 004 reflection, as expected from the slope integral analysis in Fig. S6. If it were the best-oriented microfibrils that carried most of the load, the area of maximum shift would be expected to curve upwards on each side of the axis. That was not the case. The line of maximum shift was straight and horizontal, showing that microfibril orientation made no difference to the crystallographic strain up to at least 15° on either side of the axis.

Another testable hypothesis is that less ordered cellulose, or any non-cellulosic polysaccharides contributing to the 004 reflection, undergo a different level of strain from crystalline cellulose. This was the initial rationale of the WANS experiments, since less-ordered cellulose and non-cellulosic polysaccharides can in principle be tagged by vapour-phase deuteration^29^. The scattering contrast underlying X-ray and neutron diffraction depends on different structural features: mainly the distribution of C and O atoms in WAXS and H or D atoms in WANS^32^. Surface cellulose (and hemicellulose) chains that are accessible to deuterium exchange^24,25^, make an increased contribution to the WANS pattern after deuteration. Nevertheless the mean position and width of the 004 reflection were not significantly different in all three types of diffraction experiment (Table S3).

**Figure 3:**
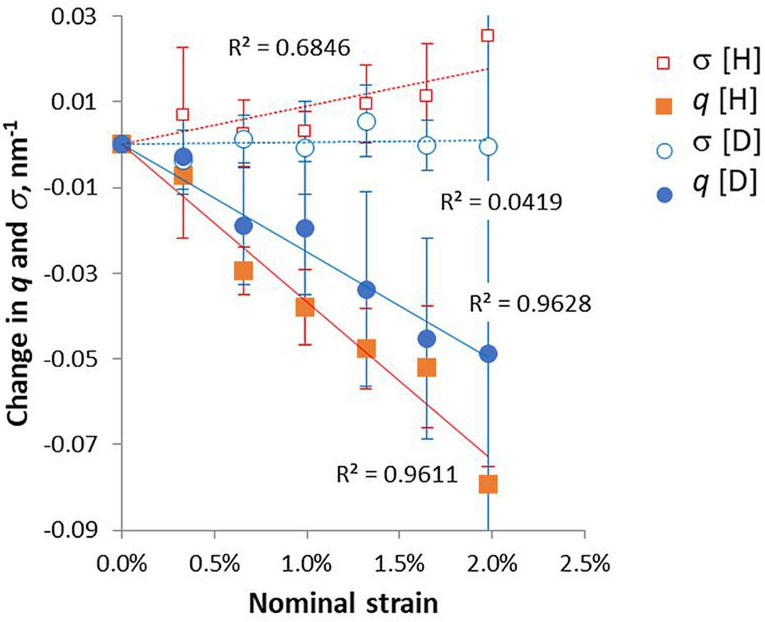
Changes in position *q* and width, expressed as standard deviation σ after Gaussian fitting, of the 004 WANS reflection under tension with and without deuteration. Mean of 6 experiments. Error bars indicate 1 SD. The difference in position *q* between the H and D forms was non-significant by ANOVA.

There was variation between WANS experiments under tension (Fig. 3). Ideally each experiment would start with a very slight pre-tension on the sample, but due to friction in the tensioning device this was not always achieved. The deuterated samples in particular showed a variable lag before they began to stretch, but this observation was probably an artefact and cannot safely be interpreted. There was no significant difference in slope between the deuterated and non-deuterated samples over six experiments at three moisture contents (Fig. 3). In the H form, the 004 reflection broadened significantly as it moved inward under tension, as in the WAXS experiments. In the D form, the effect of tension on the width of the reflection was non-significant (Fig. 3).

The approximate position and width of the 004 reflection were recovered when the tensile stress was removed (Fig. S7), despite substantial irreversible macroscopic elongation of the sample (Table S1).

### Minor axial reflections in WAXS and WANS

**Figure 4:**
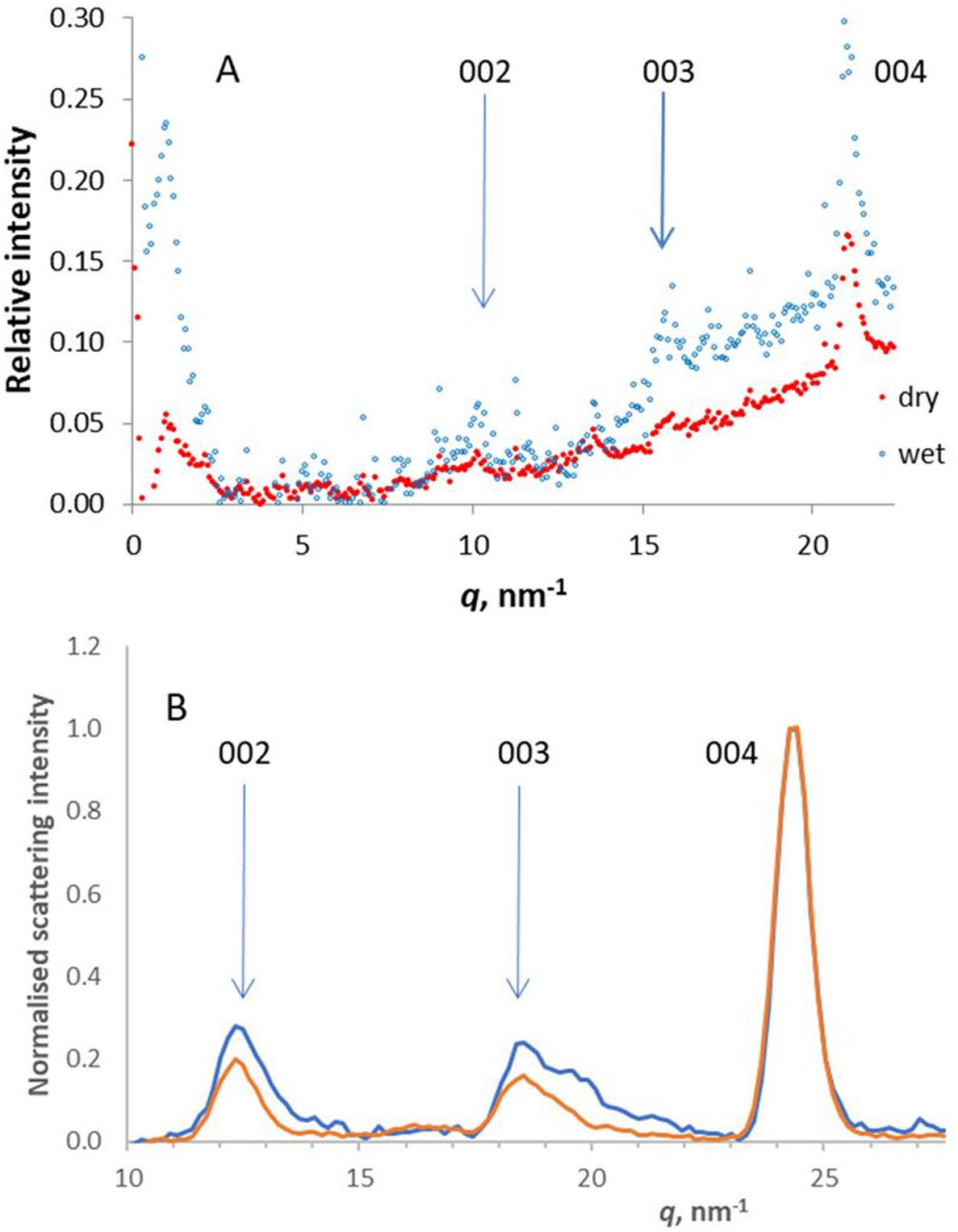
Minor axial reflections. (**A**) WANS axial profiles from wet and dry Sitka spruce wood, showing the 002, 003 and 004 reflections (*n* = 1). (**B**) WAXS axial profiles from wet (blue) and dry (red) Sitka spruce wood, normalised on the intensity of the 004 reflection (*n* = 4). By one-way ANOVA (*n* = 4) the increased relative intensities of the 002 and 003 reflections in the hydrated state were significant (002: *F* = 39.2, *P* < 0.001; 003: *F* = 6.3, *P* < 0.05).

In the X-ray and neutron diffraction patterns from cellulose Iβ the 001 and 003 reflections are absent and the 002 reflection is very weak^32^. Modelled WAXS patterns from small disordered microfibrils include the 002 and 003 reflections at very low intensity^35^. However in the WANS patterns from spruce wood (Fig. 4A), hardwoods^29^ and bamboo^27^ these reflections were clearly visible at 10–25% of the maximum 004 intensity after deuteration, but not without deuteration. In spruce wood, the 002 and 003 WANS intensity increased on hydration (Fig. 4A). The 002 and 003 reflections were also present in the WAXS patterns from spruce wood, where they increased slightly in intensity on hydration (Fig. 4B).

**Figure 5:**
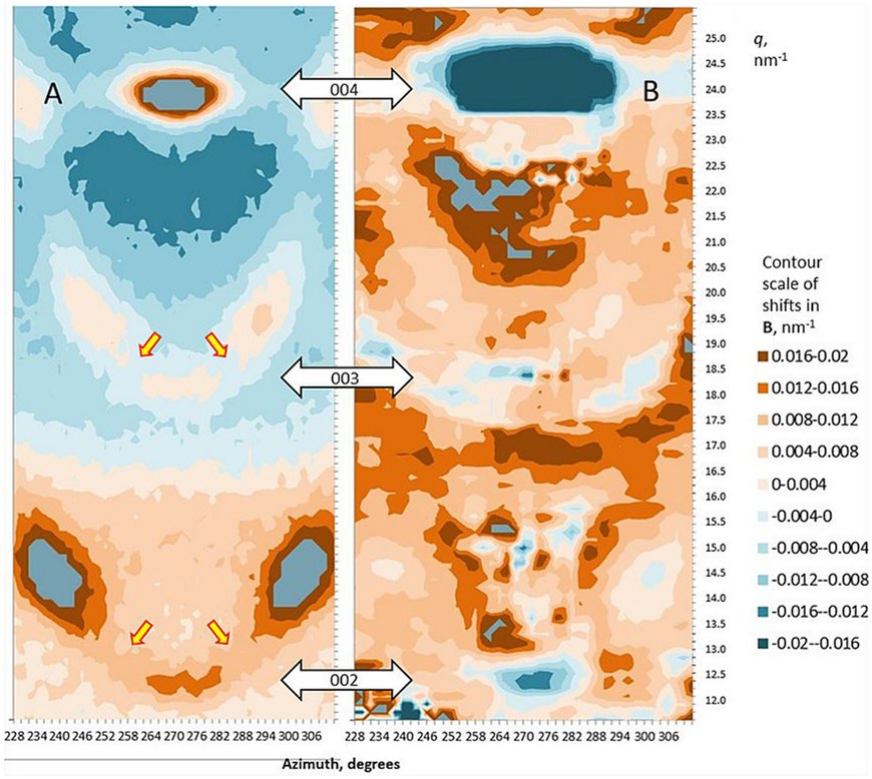
Correlative shift map of the axial region of the WAXS patterns under tension (right) compared with scattering intensity (left) in polar co-ordinates so that each layer line (yellow arrows) curves upward from the fibre axis at 270°. The scattering intensity scale and the contour scale of the shift map differ by a factor of 5 from Fig. 2 to show the weaker features. Average of four experiments. It is not practicable to indicate variation from the average in a 2D plot but the reproducibility can be gauged from Figs. 1, S4, S6 and S9 which include estimates of variability in 1D. The 2D shift correlation was carried out in discrete regions around each pixel of the diffraction pattern (2° of azimuth and *q* = 0.15 nm^−1^), so that there was some smearing of the shifts and they were artificially smaller in regions of low scattering intensity. Quantification of the shifts in 2D was therefore not uniform and the contour scale is approximate.

The axial unit cell dimension estimated from the 002 and 003 WAXS reflections was 1.4% shorter than that measured from the 004 (Fig. S8A) with or without tilting the sample. The azimuthal width of the 002 and 003 reflections was approximately 12° (Fig. S8B), expressed as σ for a fitted Gaussian function; twice as wide as the main part of the 004, 200 and other major reflections, although these also contained a minor broad component (Fig. S8C). The increased azimuthal width might imply a contribution from diffuse scattering. Figure 5 shows that diffuse scattering intensity was present along the second and third layer lines, particularly along the third layer line. Diffuse layer-line intensity is commonly associated with aligned but laterally disordered fibres^36^. However even separating out the diffuse scattering by correlative shift mapping (see below) the azimuthal width and unit cell length for the discrete part of the 002 reflection were greater than for the 004.

The diffuse components of the 002 and 003 reflections therefore should represent domains that are less ordered than the rest of the cellulose microfibrils. By comparing relative shifts in these reflections with those in the 004 reflection, it should be possible to detect any differences in molecular strain between crystalline cellulose and these less ordered domains, within a single experiment. In the WANS experiments the signal/noise ratio was insufficient to locate the radial centres of the minor axial reflections with enough precision to estimate shifts under tension: this was only possible, although challenging, in the WAXS experiments and only without tilting.

The 002 and 003 WAXS reflections moved inward under tension as did the 004 reflection. When averaged over five experiments in which the correlations of the 002, 003 and 004 *q* values to nominal macroscopic strain exceeded 0.8, the slope ratio 002 : 004 was 1.04 (SD = 0.25) and the slope ratio 003 : 004 was 0.88 (SD = 0.13), both slope ratios being non-significantly different from unity. Thus the increase in axial dimension under tension, measured from the 002 and 003 reflections, did not differ significantly from the increase in axial dimension measured from the 004 reflection. This analysis is based on the *q* values at the azimuthal centre of each reflection, which contains a contribution from scattering intensity along the second and third layer lines. Using the correlative shift mapping approach, these scattering contributions were disentangled as shown in Fig. 5.

The shift at the 002 reflection as shown in Fig. 5 was largely restricted to the discrete reflection itself. Similarly, most of the 003 shift was concentrated at the discrete 003 reflection with any shift difficult to distinguish from noise in the arcing region of diffuse scattering intensity along the third layer line on either side. In the region of the 003 reflection there was also an arc of increased shift at about *q* = 18 nm^−1^, but the scattering intensity there was too low to represent any major component of the wood material. These observations imply that the structural features that gave rise to the discrete 002 and 003 reflections stretched together with the rest of the microfibril lattice under tension, but there was little stretching of the disordered chains responsible for the diffuse layer-line scattering.

### Changes in microfibril spacing estimated by SANS

Deuterated spruce wood gives two overlapped coherent SANS peaks: a major peak with a characteristic spacing of 3.0 nm when dry^9^, corresponding to close-packed 3 nm microfibrils and widening on hydration. A minor 3.7 nm peak has been interpreted as microfibrils separated by bound xylan^14^. To determine if microfibril spacings changed under stress, SANS data were collected during a stress-relaxation experiment on deuterated spruce wood (Fig. S9). There was no detectable change in the position of the main coherent SANS peak under tension or stress relaxation, implying that the characteristic centre-to-centre spacing of uncoated microfibrils remained unchanged, even though the microfibril diameter would have been expected to decrease under tension according to the Poisson ratio of about 0.5 for cellulose^8^. The position of the minor SANS peak could not be estimated with enough precision for comparison.

### FTIR bandshifts under tension

Stretching a polymer chain leads to elongation and weakening of the linkages that hold the chain together, and a consequent shift in the frequency of their stretching vibrations that can be measured by FTIR spectroscopy^30^. This approach has been used to distinguish the mechanical contributions of different polymers in wood^22^.

As applied to cellulose, mid-range FTIR spectroscopy is well established^30^. However, a problem in applying this method to wood is the limited extent to which the spectra have been assigned, particularly to non-cellulosic polymers in the 1000–1200 cm^−1^ region where most vibrational modes are highly coupled and overlap strongly^37^. A more comprehensive set of assignments was built up by comparing transmission FTIR spectra from spruce samples that had been fractionated by chlorite delignification, alkali extraction and partial acid hydrolysis (Fig. S10), or deuterated (Fig. S11) and through comparison with literature data^24,25,38–40^ from similar fractionation experiments and FTIR measurements on the isolated polymers (For details see SI). Crystalline and surface cellulose were readily distinguished by deuteration but there were more limited opportunities for unambiguous measurement of glucomannans and xylans. It should be noted that the longitudinally polarised band centred around 1160 cm^−1^, which has been widely used for assessing the orientation and stretching of crystalline cellulose^8,41^, also contains overlapping contributions from less ordered cellulose, xylans, glucomannans and even lignin.

A one-dimensional form of the correlative shift approach described above was adapted for plotting shifts in the FTIR spectrum under tension. With the additional assignments for the crowded 1000–1200 cm^−1^ spectral region this approach allowed more information on bandshifts to be extracted than has hitherto been possible. In this spectral region the clusters of split peaks comprising tensile difference spectra or dynamic spectra^42,43^ are too complex to be readily interpreted and baseline subtraction is too imprecise for bandshifts to be estimated by the difference integral method.

**Figure 6:**
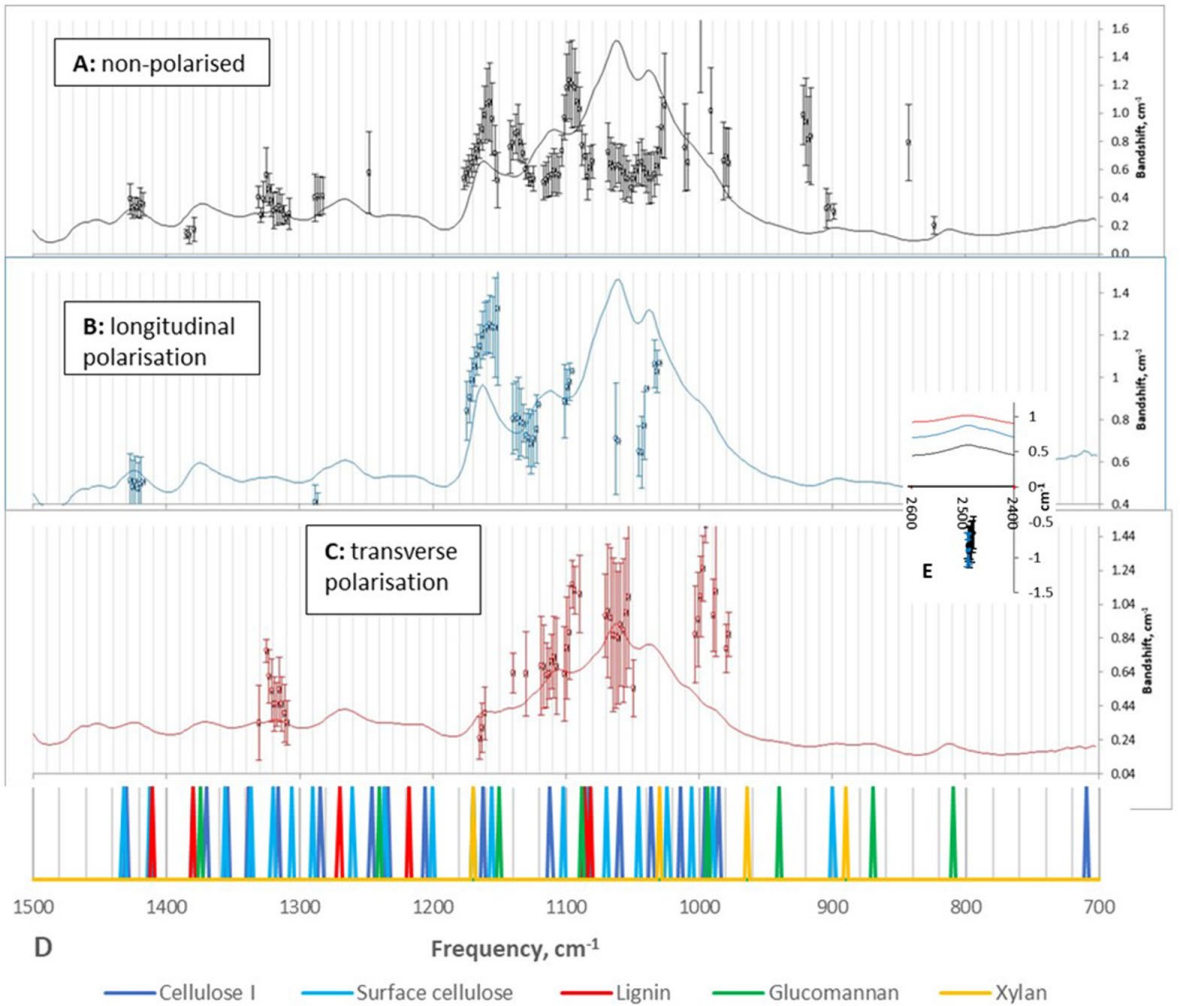
Above: FTIR spectra (solid lines) and spectral plots of bandshifts (open circles) in the non-polarised (**A**), longitudinally polarised (**B**) and transversely polarised (**C**) spectra of spruce wood elongating under tension from zero to 2.3% mean strain. Data from six experiments with error bars indicating 1 SD. For the spectra the vertical scale is absorbance. (**D**) bar chart of band assignments. Inset (**E**): negative bandshifts in the O-D stretching region around 2500 cm^−1^, with neutral and longitudinal polarisation coded in the same colours.

Detectable bandshifts were associated with all forms of cellulose and to a smaller extent with glucomannans (Fig. 6), although overlapping bands assigned to different polymers were a common complication. Bandshifts associated with xylans were not consistently detected, because the regions of the spectra where xylan signals are expected (e.g. 940–950 cm^−1^) were crowded or had little slope so that correlation coefficients were low. However O-D stretching intensity at 2494 cm^−1^ in alkaline-deuterated samples is now thought to arise from cellulose chains underlying bound xylan^14^. In this region negative bandshifts were observed (negative because O-D stretching vibrations move to higher frequency when the associated hydrogen bonds elongate^14^). Their magnitude was slightly less than the 1162 cm^−1^ bandshifts (Fig. S11), consistent with similar magnitude to the corresponding O–H stretching bandshifts for non-deuterated cellulose^22^ after correction for the isotope effect on frequency. This comparison implies that microfibril segments with and without bound xylans elongated to similar extents. Bandshifts associated with lignin were either non-significant or unconfirmed due to overlap (Fig. 6).

Where the signal/noise ratio was sufficient, bandshifts were approximately linear with strain up to the point of fracture (Fig. S12). In a few places bandshifts were associated with dips in the spectra, e.g. around 930 cm^−1^ and 1200 cm^−1^. These were probably an artefact of the correlation-based approach where a dip was adjacent to a shifting band. Significant bandshifts were generally similar in the non-polarised, transverse and longitudinal spectra (Fig. 6), with exceptions where longitudinally and transversely polarised bands overlapped. For longitudinally polarised bands the shift was not always significant in the transverse spectra and vice versa. The shift of the band around 1162 cm^−1^, formerly assigned to glycosidic C–O–C stretching of cellulose, decreased between 1160 and 1170 cm^−1^ (Fig. 6), consistent with heterogeneous elongation of cellulose chains as observed through broadening of the 004 WAXS/WANS reflection, but this interpretation cannot be safely drawn from the FTIR experiments due to the contribution of other polymers to the complex 1162 cm^−1^ band. It should not necessarily be assumed that equal elongation of conformationally different cellulose chains, e.g. in the interior and surface regions of any microfibril, would give rise to equal bandshifts: apart from possible differences in vibrational coupling, cellulose chains with fewer intramolecular hydrogen bonds may elongate by straightening more than by stretching of the glycosidic linkage or the pyranose ring^8,44^.

FTIR bandshifts were recorded during stress-relaxation experiments (Fig. S13), in an attempt to test the hypothesis that bridging elements involved in stress transfer between microfibrils would stretch more under tension, and would relax more completely when the sample was held at constant length and the stress decayed with biexponential kinetics (Fig. S1).

The cellulose stress-relaxation bandshifts associated with crystalline and disordered cellulose (Fig. S13) generally mirrored those during the initial stretching phase, with smaller magnitude and opposite sign, consistent with the microfibril stress relaxing simultaneously with the macroscopic stress. The tension-induced shift in the 1162 cm^−1^ band, decaying during stress relaxation (Fig. S14), was well fitted by a dual-exponential kinetic function with the same fast and slow time constants as were measured for the decay of macroscopic stress (Fig. S1), although the fraction of the bandhift that relaxed with these kinetics was smaller than the fraction of the nominal macroscopic stress.

Delignified and vapour-deuterated samples were used to clarify the stress-relaxation behaviour of the non-cellulosic polymers (Fig. S13). The 1087 cm^−1^ band assigned to glucomannan shifted on stretching but not significantly on relaxation. It was not possible to distinguish the 1087 cm^−1^ contributions of cellulose-bound and unbound glucomannans, so these were averaged. Bandshifts associated with the signals from the acetyl substituents on glucomannan, which are not expected to be load-bearing, were absent or inconsistent. After delignification, however, small positive relaxation bandshifts were observed for the 810 cm^−1^ glucomannan signal and the 1735 cm^−1^ acetyl signal (Fig. S13). Lignin bandshifts on stretching were generally non-significant but very small negative bandshifts, close to the limit of detection, were observed for some lignin bands during the relaxation phase.

The bands showing characteristic stretching and relaxation shifts in these experiments (Fig. S13) closely paralleled the split peaks in the synchronous and asynchronous spectra, respectively, obtained by dynamic spectroscopy^41^, implying that relaxation phenomena on the timescale of dynamic spectroscopy (10^–1^ s)^42^ resemble those on the 10^2^ s–10^3^ s relaxation timescale described here.

Creep led to very small, generally non-significant, bandshifts for all identifiable polymers after the initial imposition of the constant tensile load (Fig. S15).

## Discussion

Our current understanding of the wood nanostructure^5^ is based heavily on NMR methods that cannot be applied under mechanical stress. Vibrational spectroscopy and scattering methods have been used to study how the nanostructure of pliant wood with high microfibril angle deforms, under stress^45^, by polymer reorientation and sliding. In wood with low microfibril angle, polymer stretching is more important than reorientation. Also, such wood is so stiff that structural changes under tension are small. The stretching of crystalline cellulose has been probed by X-ray scattering, but not the stretching of disordered polymers whose contribution to scattering patterns is less understood. Vibrational bandshifts under tension have also been used, particularly in the 1162 cm^−1^ FTIR band assigned to glycosidic C–O–C stretching mode in crystalline cellulose, but the ring and glycosidic C–O–C stretching modes for other polymer chains are hidden in the complex 1000–1130 cm^−1^ spectral region where many bands overlap and are hard to assign.

The present study included a number of innovations to overcome these problems: correlative shift analysis for extracting small shifts from complex spectroscopic and scattering datasets; X-ray and neutron scattering under tension, making use of the minor axial reflections that originate from structural disorder in the microfibrils; and more detailed FTIR assignments applied to time-dependent stretching experiments.

One aim of these experiments was to determine whether crystalline and disordered microfibril components stretched together^22^. FTIR bandshifts, *q* shifts after deuteration in WANS and *q* shifts in the discrete 002 and 003 WAXS reflections all indicated concerted stretching. The identity of the structural components responsible for the discrete 002 and 003 reflections is uncertain and simulation^7,46^ of diffraction by wood microfibrils would be desirable before conclusions are drawn about their distinctive position and azimuthal width, but their broad azimuthal distribution is shared with the non-coherent SANS intensity and a portion of the 004 and other major WAXS reflections. They appear to represent microfibril domains that are less ordered, less aggregated and therefore more accessible to moisture and deuteration.

Where wood under tension has been studied by WAXS, a common observation is that the crystallographic strain, measured from the 004 reflection in cellulose, is less than the macroscopic strain. The ratio varies in published experiments, from 0.1 or less^20^ to near unity^34^ due at least partly to the ‘thin sample’ effect^18^. It averaged about 0.25 in our WAXS experiments and 0.1 in WANS, the difference being partly due to relaxation during the long measurement times (> 1 h) in WANS.

It follows that the cellulose microfibrils must slide past one another as well as stretch. This observation raises several questions. What is the sliding mechanism, and what structural features resist it? Is it the same as the ‘Molecular Velcro’ mechanism suggested^2,17^ for wood with high microfibril angle? Where does it occur, within or between the microfibril bundles (macrofibrils) now known to occupy the main S2 layer of the softwood cell wall^12–14^? Within the microfibril bundles, SANS experiments reported here and elsewhere^14^ show that there is direct or hydrated contact between microfibril surfaces, some of which are xylan-coated^5^. The space between microfibril bundles is occupied by lignin^47^ and acetylated glucomannan^14^, which would be expected to deform if resisting shear.

We were not able to answer all the above questions but our findings narrow the range of possibilities. One hypothesis that can be excluded is that each microfibril contains long ordered and disordered domains in series, with stretching largely restricted to the disordered domains. The FTIR experiments and the WAXS and WANS data, including the WAXS data on the minor axial reflections, did not show that disordered cellulose stretched more than crystalline cellulose.

We were able to test the hypothesis that tensile elongation of softwood cell walls is resisted by non-cellulosic polymers bridging between microfibrils or, more likely, between macrofibrils. Viscous slippage of hemicellulose bridges (considered then to lie between microfibrils rather than between macrofibrils) was suggested to explain the non-elastic component of tensile deformation in the molecular Velcro hypotheses for high-MFA wood^2,17^. Consistent with this hypothesis, the cellulose microfibrils returned to approximately their original unit cell length as measured by WANS, simultaneous with an irreversible increase in macroscopic sample length. If non-cellulosic bridging polymers were responsible they might be expected to show considerable extension or reorientation observable by FTIR, fully reversed on stress relaxation. Non-cellulosic polymers behaving in that way were not identified. Some of the glucomannans appeared to stretch and relax with the cellulose, perhaps because they were bound to microfibril surfaces^5^. There were only slight signs of glucomannan and lignin perturbation during relaxation and creep, consistent with earlier observations on shorter timescales by dynamic FTIR^48,49^. The diffuse scattering on the second and third layer lines suggested that structures with partial orientational order but disordered packing did not stretch on the timescale of our experiments. This fraction would probably include at least parts of the lignin-glucomannan matrix between the macrofibrils.

**Figure 7:**
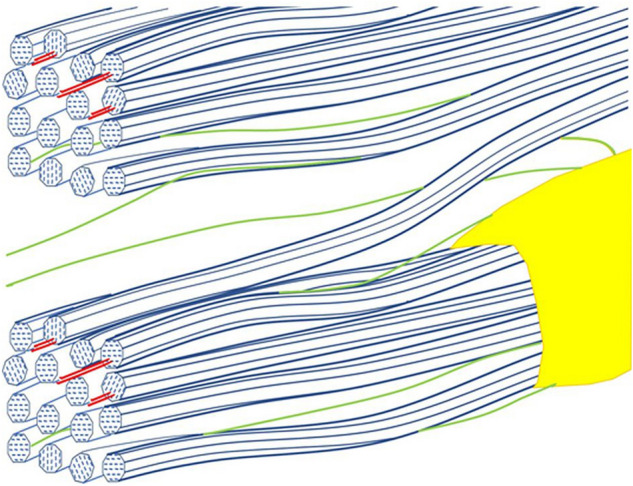
Diagrammatic view of a portion of the spruce cell wall, with two cellulose macrofibrils (blue) containing xylan (red) that is partly bound to cellulose surfaces. The macrofibrils are separated by a matrix composed largely of glucomannan (green) and beaded lignin (yellow). Two macrofibrils are shown bridged by a single microfibril, suitably positioned to transmit shear load between the macrofibrils if their position within the larger-scale structure leads to their axial stress being unequal. Within each macrofibril the microfibrils are shown as held together by non-covalent forces but the right-handed microfibril twist^52^ prevents attachment zones from being axially continuous.

The observation that microfibrils stretched to varying extents, from near zero to about twice the average crystallographic strain (but less than the wood itself), implies that there are discrete attachment points of some kind between microfibril surfaces; and that the part of a microfibril between two such attachment points can bear a large or a small fraction of the overall tensile stress. We looked unsuccessfully for features discriminating between the stressed and unstressed microfibril domains, including orientation, crystalline disorder, hydration and the presence of bound xylan. While this search was not exhaustive, it suggests that the heterogeneity of local stress does not arise from structural variation between microfibril segments, but from their location within the disordered, anastomosing topology of microfibril aggregation^50,51^. For example (Fig. 7) a microfibril segment (cross-section 10 nm^2^) that bridges between a loaded and an unloaded macrofibril (cross-section > 100 nm^2^) would carry more stress than the macrofibrils that it connects.

Without ruling out matrix shear, it seems worthwhile to look at the possibility that some of the attachment points comprise direct contact between two microfbrils^53^, contacts capable of sliding under shear stress or breaking and re-forming in a different place^17^ as has been suggested for primary cell walls^54^. The hydrated non-cellulosic polymers might then have a role in modulating such direct microfibril-microfibril contacts: that is, in keeping microfibrils apart rather than joining them together.

The SANS experiments gave some insights into the abundance of direct contact between microfibrils, with or without intervention of water that might act as a lubricant. The characteristic centre-to-centre spacing of microfibrils uncoated with xylan varies from about 3.1 nm in the dry state to 3.9 nm at full hydration^9^ and did not change under tension. These spacings are not averages but a function of a distribution whose lower bound is at contact^9^. They would accommodate one or more interstitial layers of water when the wood cell wall is moisture-saturated, but not at the lower moisture contents where unequal microfibril stress and time dependence are already observed. A mix of water-accessible and water-inaccessible interfaces has been demonstrated by NMR^55^ and deuteration^56^ experiments on wood and on pulps consisting mainly of cellulose. In the present experiments on spruce wood the wider microfibril spacings where a xylan chain intervenes could not be measured with precision, but in hardwoods these pairs of microfibrils are not prised apart by water^29^.

Different microfibril surfaces could interact in different combinations. The SANS experiments demonstrated contacts between hydrophilic microfibril faces, where deuteration provided neutron scattering contrast. Shear between hydrophilic faces would depend on hydration and on the structure of the interstitial water. Shear might alternatively be facilitated along an interface between the hydrophobic [200] faces of a pair of microfibrils where the interfacial attraction is more delocalised, like internal shear between sheets of chains when a microfibril bends^57–59^. In softwoods the [200] faces are not now considered to occupy a very large fraction of the microfibril surface^60^.

These observations highlight difficulties that have always existed in the study of wood under stress. In particular, thin sections of low-MFA conifer wood, with dimensions under about 1 mm, stretch with lower moduli than bulk low-MFA wood and with a larger time-dependent, partially irreversible contribution to their elongation^18^. The thickness-dependent quantitative differences shown in Fig. S1 and Table S1 mean that caution is needed in extrapolating deformation mechanisms, especially from the 20 µm FTIR sections, to thick wood samples. The accentuated slippage between microfibrils is much easier to study in thin samples but its mechanism may not be identical. However these experiments would have been impracticable on thicker samples due to infrared opacity (FTIR), or self-absorption (WAXS, WANS)^18^.

Wood cell walls under tension absorb moisture^33^. Some of the absorbed water may penetrate between the microfibrils because each microfibril contracts laterally^8^ without any change in spacing. Water may also move into the matrix between the macrofibrils^61^. Water sorption kinetically resembles the viscous component of stretching^62^, and the time and moisture dependence of Poisson ratios^63,64^ would be consistent with coupling between tensile deformation and internal redistribution of water.

Although wood is stiffer and less tough than other non-mineralised biological materials, its time-dependent deformation is likely to absorb energy similarly and resist fracture, both in the living, hydrated state^3^ and when dried as a constructional material. Understanding the nanoscale mechanisms will help us to extract optimised performance from softwoods and to design new materials, made from wood or otherwise^65,66^, with enhanced strength and manufacturability.

## Materials and methods

### Materials

Mature earlywood from Sitka spruce (*Picea sitchensis* Bong. (Carr.)) was prepared as described previously^8^. Longitudinal-tangential sections approximately 2.5 mm wide and 0.5 mm thick were excised with a razor blade and attached to aluminium tabs, drilled for attachment to the tensile test rig, with epoxy resin in a purpose-built jig, leaving a gauge length of 38 mm, as described^8^. For neutron diffraction, three such sections were mounted side by side on the same pair of tabs. For FTIR, microtome sections with nominal thickness 20 µm, 1 mm wide, were prepared as described^8^ and attached with cyanoacrylate to the fixed and sliding ends of a screw-driven, humidity-controlled tensiometer.

### Statistical analysis

Significant differences (*P* < 0.05 unless otherwise stated) were estimated by one-way or two-way ANOVA with two-tailed F tests and *n* as stated for each comparison.

### Tensile testing

Longitudinal-tangential sections were attached by their aluminium tabs to pins fitted to the jaws of a Tinius Olsen H1KS tensile testing machine with a 250 N load cell (Tinius Olsen 6 Perrywood Business Park, Honeycrock Lane, Salford, Surrey RH1 5DZ, England). A simple non-circulating humidity chamber was fitted enclosing the sample for measurements in the dry state or wet at above 30% moisture content, the approximate fibre saturation point. Load-deformation curves were recorded at a constant speed of 1 mm per min. The tensile modulus was calculated from the slope of the linear region and the sample dimensions measured dry with a screw micrometer. For stress-relaxation measurements the decaying load was measured manually at 30 s intervals. Strain was determined from the input crosshead position and all strain measurements were corrected for machine deflection using the load–deflection curve measured with a 15 × 2 mm aluminium bar substituted for the sample.

### WAXS with tensile stress

Samples were stretched at ambient temperature and humidity (∼50% relative humidity) on a purpose-built screw-driven tensile test rig fitted to the goniometer head of a Rigaku R-axis/RAPID image plate diffractometer with a Mo Kα radiation (λ = 0.07071 nm) source, as described^8^. The sample was set with its axis either vertical or tilted at 7° away from the beam, the tilt angle being calculated to record the full width of the 004 reflection in one half of the diffraction pattern. When the sample was not tilted, strain was measured directly from the gauge length of the sample, i.e. its free length between the attachment tabs, using a digital micrometer. With the sample tilted, strain was measured from the pitch of the driving screw thread (0.4 mm) and the leverage (× 5 or × 10). Collection and adjustment time for one data point in each stretching experiment was approximately 20 min. Rigaku CrystalClear version 1.4.0 and AreaMax version 1.1.5 (Rigaku Inc., The Woodlands, TX) were used to collect the X-ray diffraction patterns and for their initial processing before conversion to polar coordinates with pixel size 2° of azimuth and 0.1° of radial position 2θ. LaB_6_ was used to calibrate 2θ and to estimate radial instrumental broadening (approx. 0.10°). Further data processing, with adjustments to compensate for beam centring and rotation between diffraction patterns within each experiment, was carried out in Microsoft Excel.

### Correlative shift mapping (CSM)

The following method was used for simultaneous mapping of radial and azimuthal shifts of both discrete reflections and diffuse features when comparing diffraction patterns from the same sample with and without stretching. The WAXS images, formatted in polar co-ordinates as above, were used without background correction. A 9 × 9 pixel window around each pixel of the diffraction pattern from the stretched sample was shifted by an adjustable δ*Χ* and δ2θ. The values of δ*Χ* and δ2θ for the window in question were then simultaneously adjusted to maximise the absolute value of the correlation coefficient *r* with the corresponding window in the diffraction pattern that had been recorded at the start of the experiment, without stretching. The resulting values of δ2θ were filtered to remove pixels with *r* below a threshold value then plotted as a contour map. These operations were carried out using a series of macros in Microsoft Excel.

### Neutron scattering with tensile stress

Neutron diffraction patterns were collected under controlled humidity with vapour-phase deuteration on beamline D19 at ILL, Grenoble as described previously for structural studies of fibrous systems^29,67–69^. A screw-driven tensile testing rig was purpose-built to fit inside the controlled-environment chamber on beamlines D19 (WANS) and D33 (SANS), on the same principle as the rig used for WAXS under tension^8^ but more compact and more robust to withstand the greater forces required because of the larger sample cross-section required to achieve adequate signal: noise in WANS. Strain was measured as for the tilted WAXS experiments. The WANS data were processed as described^29^. Radial instrumental broadening was similar to WAXS although azimuthal broadening was greater. A WANS tensile experiment with 6 stretch levels typically required 12 h of beamtime. The SANS data were collected and processed as described^14^.

### FTIR spectroscopy under tensile stress

Spectral series were recorded from longitudinal-tangential sections during progressive elongation, stress-relaxation and creep experiments, in some cases with vapour-phase^9^ or partial internal deuteration^8^ in a purpose-built, screw-driven extensiometer. Strain was determined directly from the pitch of the screw thread (0.4 mm). Bandshifts were extracted from the spectral series by correlative shift analysis, an approach analogous to the two-dimensional CSM described above. Within a seven-point (14 cm^−1^) moving window, the spectrum recorded under stress was shifted in frequency by δ*v* and correlated with the initial spectrum, adjusting δ*v* to minimise (1 − *r*^2^). Because flat regions of the spectrum gave low correlation coefficients and spurious shifts the output data were filtered using the gain factor (1 −* r*^2^)_δ*v*_ / (1−*r*^2^)_(δ*v*=0)_. A gain factor of 10 or 20 was considered to be significant depending on the experiment. The raw spectra with no baseline correction or normalisation were used directly as in the CSM procedure, minimising artefacts.

## Supplementary information

Supplementary Information 1.

## Abbreviations

FTIR: Fourier-transform infrared
SANS: Small-angle neutron scattering
WANS: Wide-angle neutron scattering
WAXS: Wide-angle X-ray scattering
NMR: Nuclear magnetic resonance
FWHM: Full width at half maximum
SD: Standard deviation
