# Supplementary material for: Nanostructural deformation of high-stiffness spruce wood under tension
Source: Sci Rep. 2021 Jan 11;11:453. doi: 10.1038/s41598-020-79676-2 (PMC7801420; doi:10.1038/s41598-020-79676-2)
Supplement: Supplementary file 1 — Supplementary Information 1. [file 41598_2020_79676_MOESM1_ESM.docx]

Nanostructural deformation of high-stiffness spruce wood under tension

Lynne H. Thomas^1^, Clemens M. Altaner^2^, V. Trevor Forsyth^3,4,5^, Estelle Mossou^3,4,5^, Craig J. Kennedy^6^, Anne Martel^3^, Michael C. Jarvis^7^

^1^Department of Chemistry, University of Bath, Claverton Down, Bath BA2 7AY, UK.

^2^New Zealand School of Forestry, University of Canterbury, Private Bag 4800, Christchurch, New Zealand.

^3^Institut Laue-Langevin, 38042 Grenoble Cedex 9, France

^4^Partnership for Structural Biology (PSB), 38042 Grenoble Cedex 9, France

^5^Faculty of Natural Sciences, Keele University, Staffordshire, ST5 5BG, UK

^6^School of Energy, Geoscience, Infrastructure and Society, Heriot Watt University, Edinburgh, EH14 4AS, Scotland, UK.

^7^School of Chemistry, Glasgow University, Glasgow G12 8QQ, Scotland, UK

Correspondence to M.C. Jarvis, email michael.jarvis@glasgow.ac.uk

Supplementary Information

Additional methods and results

**Tensile properties of Sitka spruce wood with low microfibril angle.** Microtome-cut wood samples with a nominal thickness of about 20 μm, less than that of a wood cell and within the range where ‘thin sample’ effects are encountered^1^, are required for transmission FTIR experiments. For WAXS and WANS the sample thickness is limited to about 0.5 mm to avoid self-absorption problems: 0.5 mm is around the ‘thin sample’ threshold^1^. At that sample thickness the large forces needed for tensile experiments present an engineering challenge, particularly within the confined space of the controlled-environment chamber used in the neutron scattering experiments. Materials that could be used in or near the neutron beam were also restricted for safety reasons. The solution adopted was to use simple, very compact stretching devices that allowed adjustment of strain but did not include transducers for measuring stress, and to carry out stress-strain measurements on the same samples offline.

The mean tensile modulus of the 0.5 mm thick samples was 9.3 +/- 2.1 GPa (standard error of the mean (SEM), *n* = 7). The 0.5 mm thick samples showed two-phase tensile stress relaxation, which was faster in the dry state (Fig. S1). The fraction of the total stress that relaxed with these kinetics was 7% +/- 1% (SEM, *n* = 7), with no significant difference between dry and hydrated states (Table S1) although hydration reduced the tensile modulus. The 20 μm FTIR samples had much larger relaxing (Fig. S1) and irreversible fractions (Table S1).


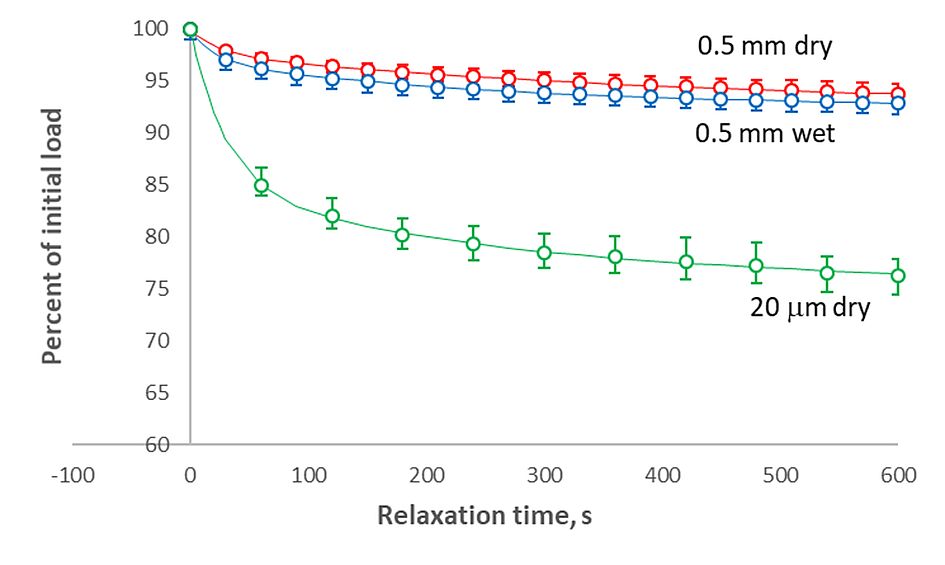


**Fig. S1.** Stress-relaxation curves for Sitka spruce samples of thickness 0.5 mm dry (*n* = 6), water-saturated (*n* = 5), as used in the scattering experiments and 20 μm as used for FTIR (*n* = 4), following an initial stress of 30 N/mm^2^. Error bas indicate 1 Standard deviation (SD). The fitted stress-relaxation curves are biexponential as in Table S1.

**Table S1.** Reversibility and stress-relaxation characteristics of 0.5 mm and 20 μm thick Sitka spruce samples in dry and water-saturated states, loaded to a mean strain of 1.03% and allowed to relax over a time period of 10 min. Mean and SD.

|  | 0.5 mm dry | 0.5 mm wet | 20 μm  dry | | 0.5 mm dry SD (*n*=6) | 0.5 mm wet SD (*n*=5) | 20 μm  dry SD (*n*=4) |
| --- | --- | --- | --- | --- | --- | --- | --- |
| Fast time constant, s^-1^ | 0.085 | 0.052 | | 0.025 | 0.017 | 0.007 | 0.002 |
| Slow time constant, s^-1^ | 0.0025 | 0.0038 | | 0.0012 | 0.0002 | 0.0002 | 0.0002 |
| Irreversible fraction | 24.9% | 22.5% | | 50.2% | 2.7% | 2.8% | n/a |
| Total fraction relaxing | 7.34% | 7.60% | | 28.0% | 1.3% | 1.7% | 25.8% |
| Fast fraction/slow fraction | 2.25 | 1.46 | | 1.41 | 0.10 | 0.18 | 0.32 |

**Polymer reorientation under tensile stress.**


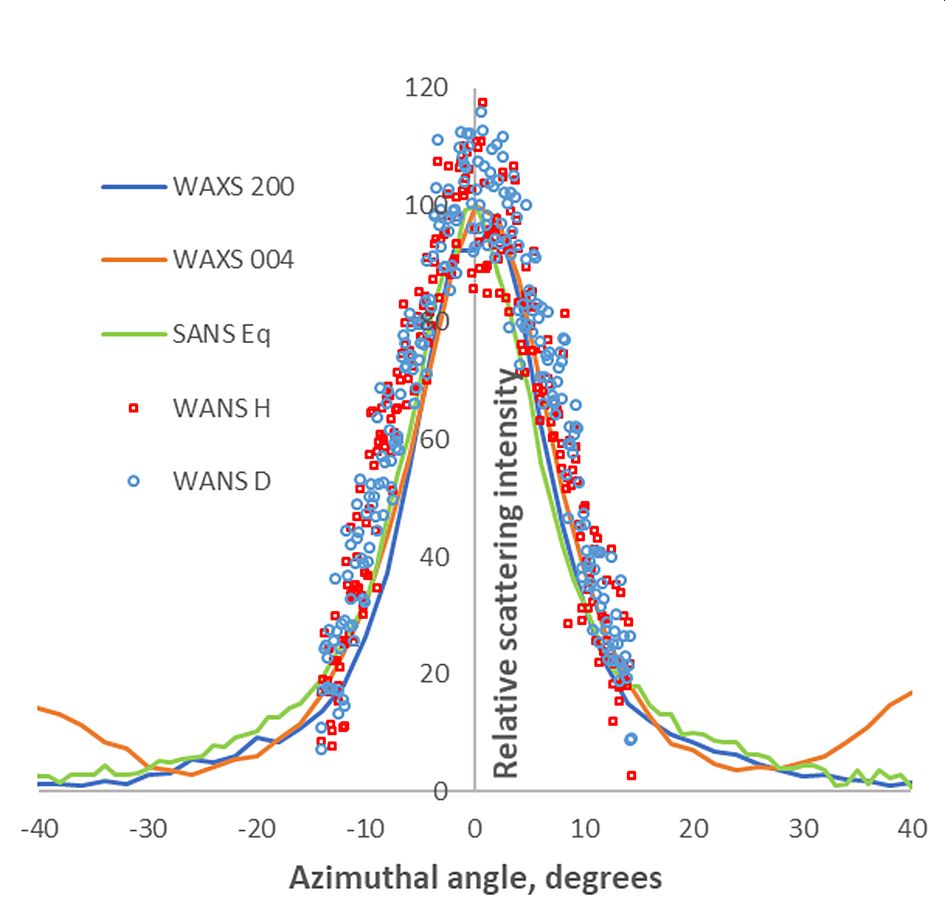


**Fig. S2.** Azimuthal scattering intensity distribution measured by WAXS (200 and 004 reflections), WANS (004 reflection) with and without deuteration, and SANS (equatorial scattering with deuteration at *q* = 2 nm^-1^). Full width at half maximum (FWHM): WAXS 12.3°, SD (*n* = 4) 0.3°; WANS H 17.9°, SD 1.6°; WANS D 17.7°, SD 1.3°; SANS 13.5°, SD (*n* = 4) 1.1°.

**
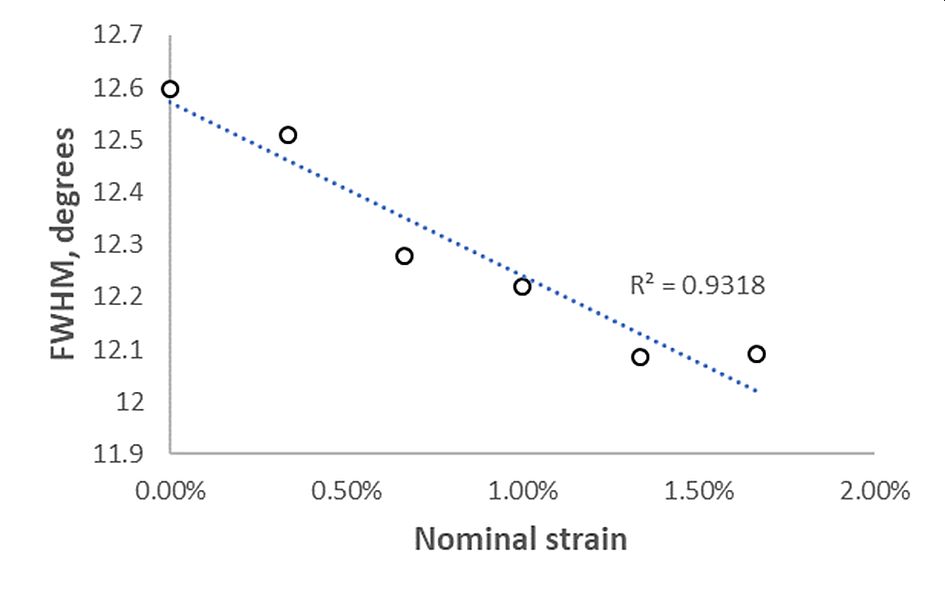
**

**Fig. S3.** Azimuthal width of the central part of the SANS equatorial intensity distribution at *q* = 0.2 nm^-1^, expressed as FWHM for a fitted Gaussian function, as affected by axial stretching in equilibrium with D_2_O vapour at 80% relative humidity. Average of the right and left halves of the diffraction pattern. By ANOVA the effect of strain was significant (*n* = 2, *F* = 20.2, *P* < 0.01).

The SANS measurements shown in Fig. S3 were derived from aggregates of microfibrils, whereas the WAXS and WANS measurements were derived from the orientation of crystal planes within each microfibril.

**Elongation of the cellulose unit cell under tension.**

A technical difficulty is that the 004 reflection from perfectly oriented crystalline cellulose can be observed only by tilting the sample to an angle that varies with the radiation wavelength, and then only in one half of the diffraction pattern. However when working with relatively short-wavelength (0.07071 nm) Mo K_α_ X-rays the 004 reflection from wood samples can be observed without tilting, at slightly reduced intensity, in both halves of the diffraction pattern^2^. This procedure gave improved precision in the measurement of the small shifts in the position of this reflection as the sample was stretched. Tilted experiments were also carried out to check that there were no resulting artefacts.

In the WAXS experiments without tilting of the sample, the sample elongation was measured directly with a digital micrometer. This was not done in the tilted WAXS and WANS experiments, where the stretching device was enclosed, and the imposed macroscopic strain is then termed ‘nominal’ because with the experimental system used it was not possible to correct accurately for a small amount of linear, elastic instrumental deflection and for end effects. The robust design of the stretching devices and reinforcement of the sample ends with metal tabs minimised these effects, but the nominal strain can be assumed to have slightly exceeded the macroscopic strain experienced by the portion of the sample that was in the X-ray beam. For this reason, inferences drawn from the ratio of crystallographic strain to nominal macroscopic strain are restricted to the WAXS experiments without tilting of the sample.

**
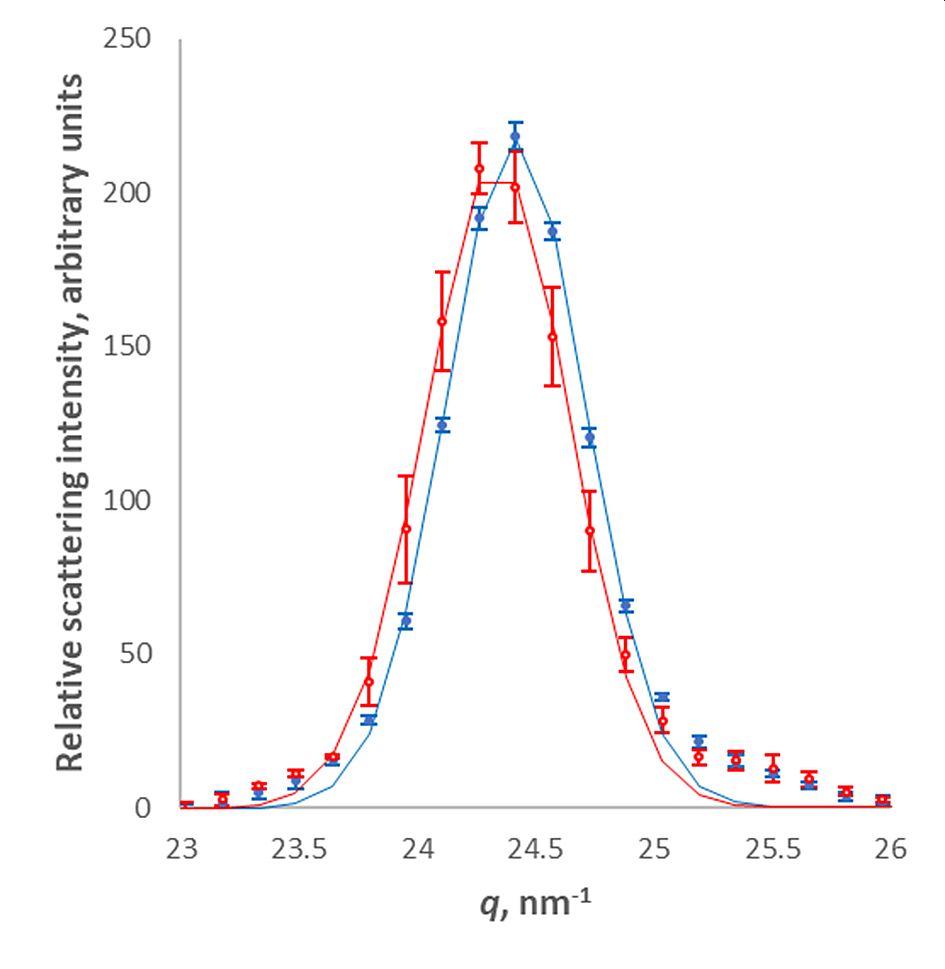
**

**Fig. S4**. Radial profile of the 004 WAXS reflection, measured without tilting the sample. Blue: zero strain; Red: 1.3% mean strain.. Four experiments on different samples: error bars indicate 1 SD.

**Change in radial width of the 004 reflection under tension.** The radial width of the 004 reflection (FWHM as *q*) without tension applied (after correction for instrumental broadening) averaged 0.56 nm^-1^SD 0.02 nm^-1^;*:* *n* = 7) without tilting and 0.52 nm^-1^ SD 0.02 nm^-1^ (*n* = 2) when the samples were tilted as in Fig. S5. The slight reduction in apparent radial width on tilting may be due to the centre of the orientation distribution being under-represented in the diffraction patterns from untilted samples.

**
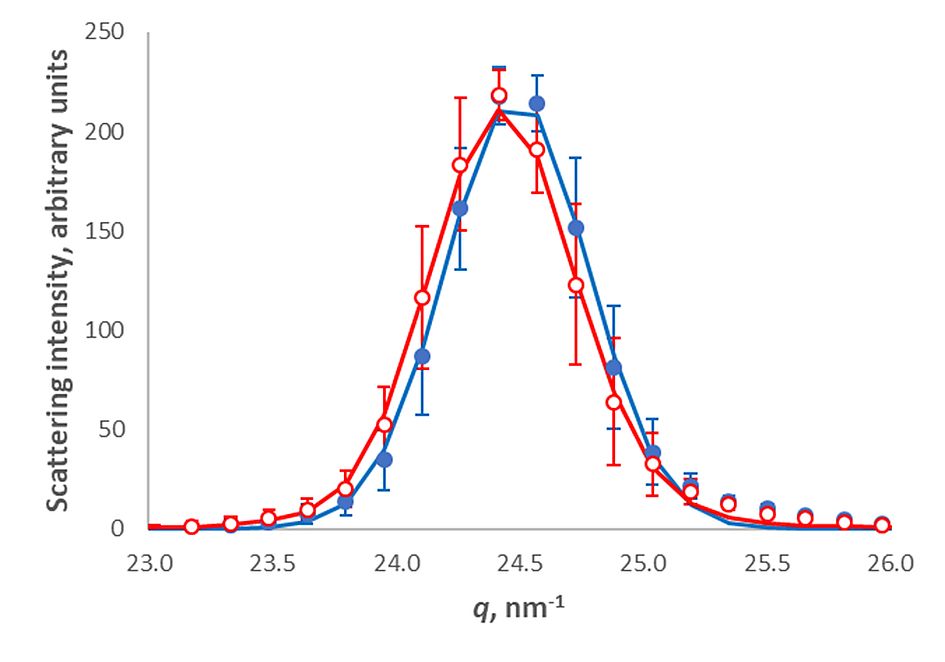
**

**Fig. S5.** Radial profile of the 004 WAXS reflection, measured with the sample tilted at 7°, at zero tensile strain (blue) and 1.08% tensile strain(red). Error bars indicate 1 SD (*n*=4).

**
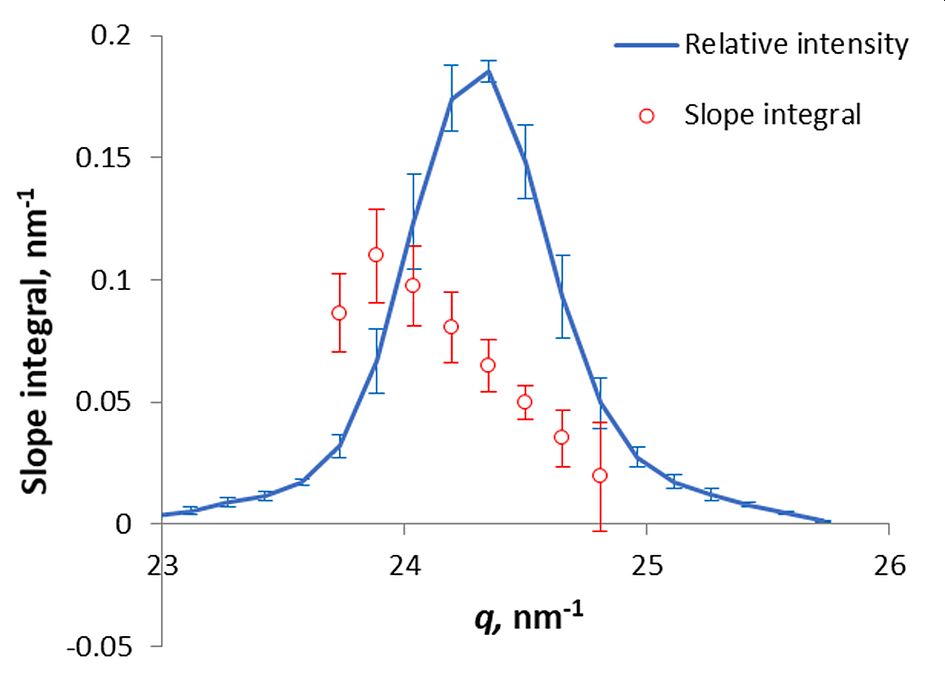
**

**Fig. S6.** Relative 004 WAXS shifts under tension, derived by the slope integral method as described^3^ at each data point and plotted across the radial width of the 004 reflection measured without tilting the sample. Error bars indicate 1 SD (*n* = 4).

**Table S3.** Radial position *q* and width (FWHM) of the 004 WANS reflection without tensile stress, based on Gaussian fitting. Mean and SD, *n* = 7. Differences between the H and D forms were non-significant (*P* > 0.05).

|  | *q*, nm^-1^ | SD | FWHM, nm^-1^ | SD |  |
| --- | --- | --- | --- | --- | --- |
| H form | 24.46 | 0.10 | 0.53 | 0.10 |  |
| D form | 24.46 | 0.11 | 0.54 | 0.10 |  |


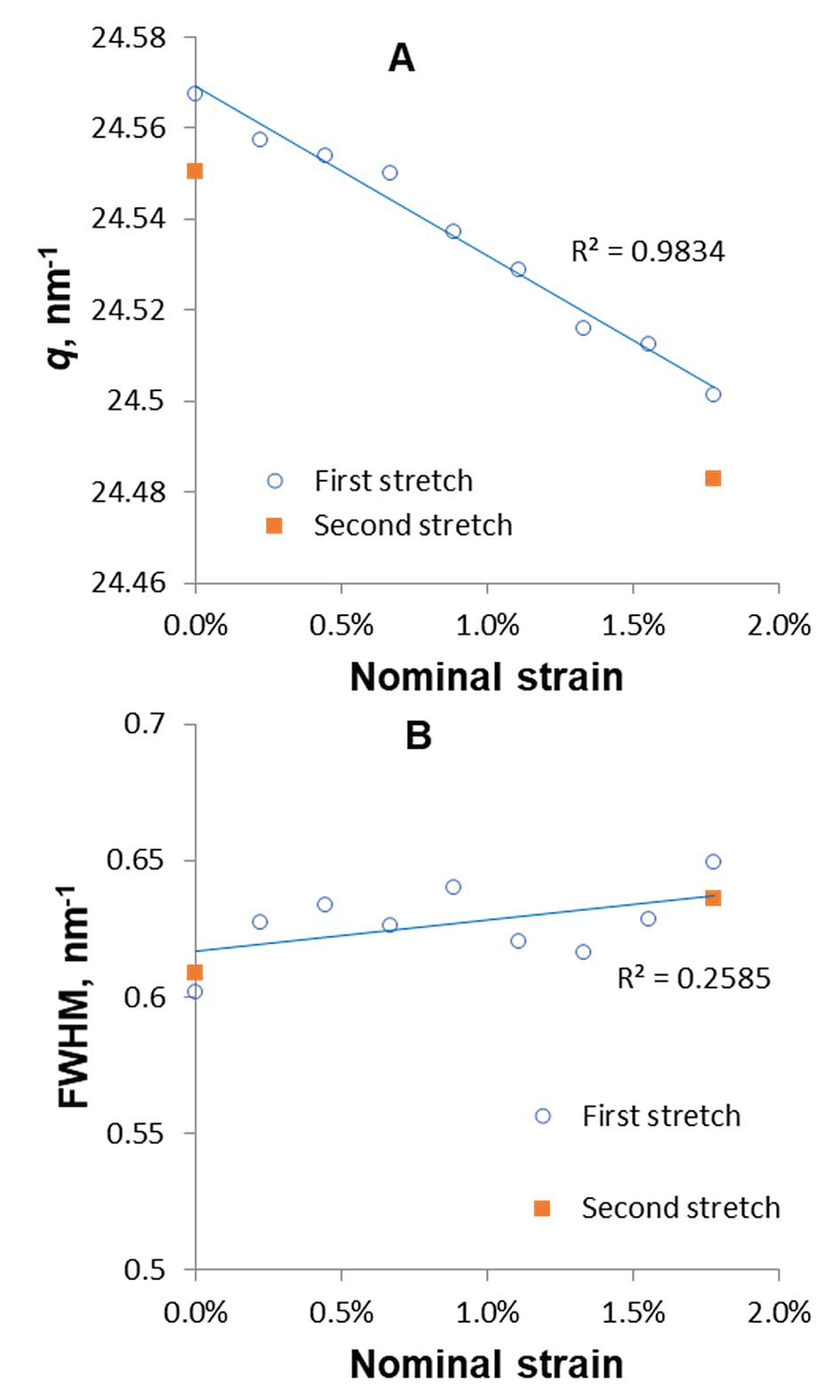


**Fig. S7.** Changes in fitted centre position (**A**) and full width at half maximum (FWHM) (**B**) of the 004 WANS reflection under tension, in two successive experiments on the same sample with vapour deuteration. The time delay between the two experiments was 1 h. Stress relaxation occurred during this time interval with the sample unloaded.

**Minor axial reflections in WAXS and WANS.**


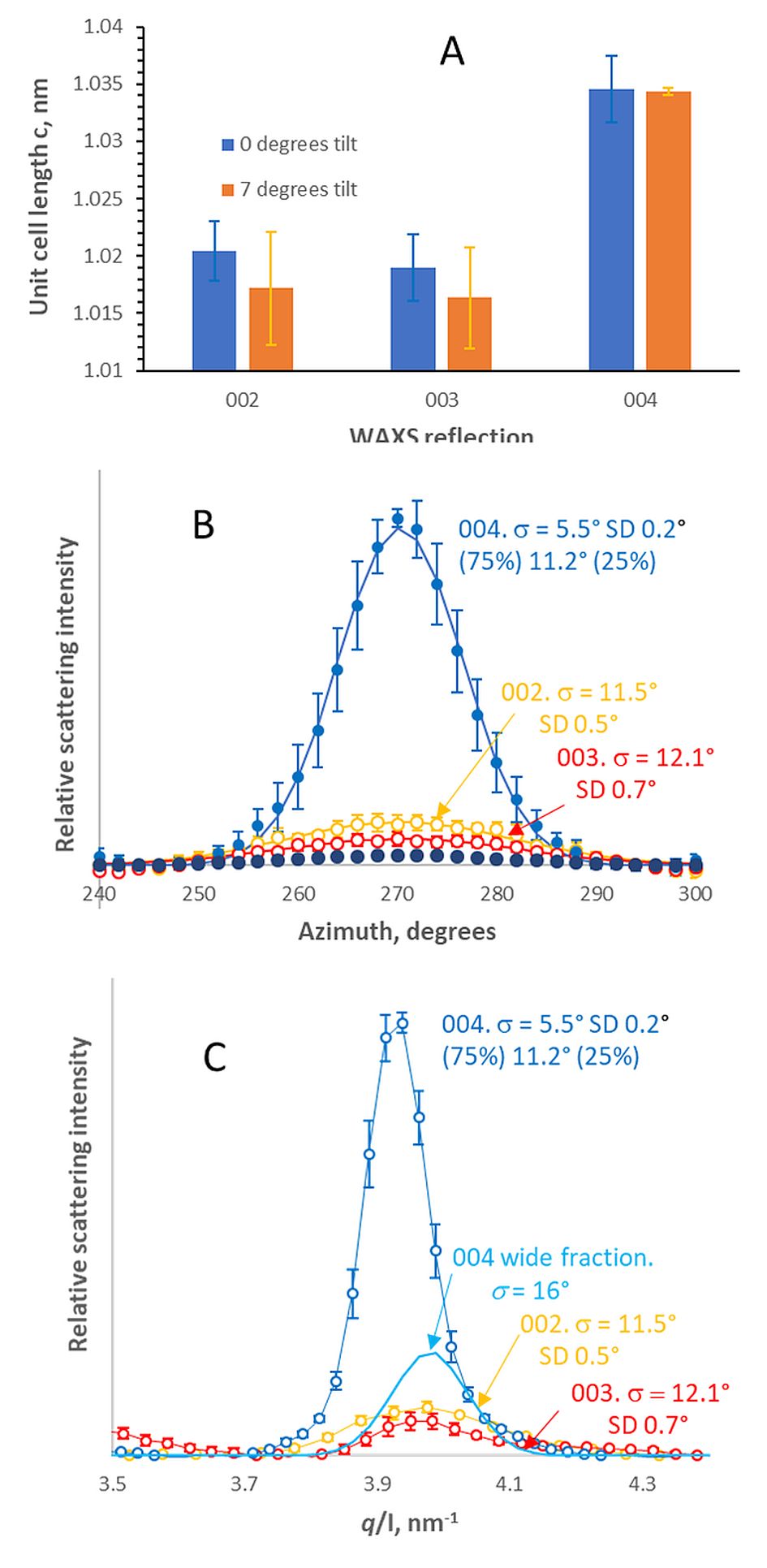


C

B

A

**Fig. S8.** Minor axial reflections. **A.** Length c of the unit cell calculated from the 002, 003 and 004 axial reflections. Mean of 6 experiments (not tilted); 2 experiments (tilted). From 2-way ANOVA the length of the unit cell was significantly greater (P < 0.001) when calculated from the 004 reflection. The calculated length of the unit cell did not differ significantly between the 002 and 003 reflections. **B.** Azimuthal intensity distributions of the 002, 003 and 004 reflections. Error bars indicate 1 SD (*n* = 4). The 004 azimuthal distribution was least-squares fitted^4^ with a dual-Gaussian function with narrow (5.5°) and wide (16.0°) components centred on the same azimuth. **C.** Radial intensity distributions of the 002, 003 and 004 reflections are plotted against *q*/*l* so that the radial scale coincides. Error bars indicate 1 SD (*n* = 4). Also plotted is the minor component of the 004 with wide azimuthal distribution, calculated from the experiment (B) in which the narrow and wide components were deconvoluted^5^.

**Changes in microfibril spacing estimated by SANS.**


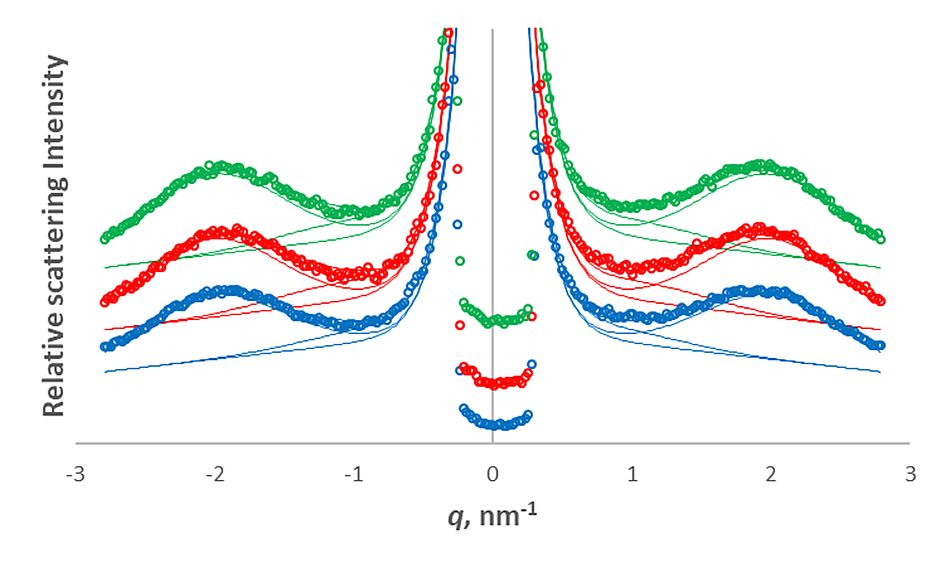


**Fig. S9.** Equatorial SANS intensity plots from Sitka spruce wood at the start of a stress-relaxation experiment (blue), under 1% tensile strain (red) and after relaxation (green). The scattering pattern is symmetrical on either side of the central beamstop (*q* = 0). The equatorial profiles were derived by 2D fitting with the coherent scattering having an azimuthal width σ = 5.6° and the wider component of the non-coherent equatorial baseline having an azimuthal width σ = 18°. The fitted major and minor coherent scattering peaks and the non-coherent scattering baseline are shown as thin lines. There was no significant difference (one-way ANOVA) in the fitted position of the major coherent scattering peak at abs (*q*) = 2.01 nm^-1^ (SD 0.02 nm^-1^), corresponding to a characteristic centre-to-centre spacing of 3.1 nm.

**FTIR bandshifts under tension.**

**FTIR band assignments.**

To assist in assigning the complex overlapping bands between 1000 cm^-1^ and 1200 cm^-1^, 20 μm Sitka spruce sections were subjected to an extraction sequence consisting of (1) delignification by the Wise procedure^6^, (2) 1M NaOH hydrolysis for 2h at 20°C followed by neutralisation with excess 1M acetic acid, to remove the xylan and a small amount of the glucomannan, deacetylating the remaining glucomannan; and (3) 1M HCl hydrolysis for 1h at 100°C followed by neutralisation with excess sodium carbonate, to remove both xylan and glucomannan. Neutralisation was followed by extensive washing with deionised water. In extraction sequence A the NaOH and HCl hydrolyses followed delignification whereas in extraction sequence B the hydrolyses were applied directly to the original material.

Spectra were compared by subtraction, using difference spectra (before extraction – extraction residue) from extractive treatments to identify the spectral contribution of the components extracted. Large baseline artefacts made simple difference spectra difficult to interpret. ‘Straightened’ difference spectra were therefore calculated by fitting a fourth-degree polynomial to a 100 cm^-1^ moving spectral window, producing a heavily smoothed function that was subtracted from the original difference spectrum to leave the flattened spectra shown in Fig. S10; the procedure is simpler than wavelet transformation^7^ but similar in effect. Comparison with published spectra of extracted polymers^8^ was helpful, particularly for lignin^9^, but was treated with caution for hemicelluloses^8,10^ due to the likelihood of distinctive conformations in the cellulose-bound form *in muro^11^*. Vapour-phase deuteration and internal deuteration under mildly alkaline conditions were carried out as described^3^. The progress of deuteration was followed over a period of 30 min (Fig. S11) giving subtraction spectra resembling those published for purified cellulose^12^. In intact wood samples, however, hemicelluloses as well as accessible cellulose surfaces are expected to deuterate.

Assignment of water-accessible cellulose components was based principally on deuteration of the intact wood cell walls (Figure S11 and^13^) and of isolated cellulose^12,14^ supported by comparison of plant celluloses with varying microfibril diameter^15,16^. Differences from the inaccessible (crystalline) forms of cellulose were small in the H form but much larger on deuteration, where comparison with internally deuterated cellulose^3^ was useful. In addition to the well-known absorption band at 1510 cm^-1^, lignin contributed more bands to the wood spectra than has been recognised, with considerable overlap with cellulose and non-cellulosic polysaccharides, consistent with the spectra of isolated lignins^9^. Lignin bands in the region below 1200 cm^-1^ arise from the aliphatic interunit linkages and would therefore be expected to show bandshifts if lignin were load-bearing, but they are too heavily overlapped by cellulose bands to be readily usable. Spectral contributions distinctive for xylans and glucomannans were generally in accord with those used in previous publications^17,18^, with one exception: bands assigned to carboxyl groups have been shown to be associated with the glucuronosyl substituents of xylans in hardwood and softwood pulps, but in spruce wood that has not been deacetylated during pulping, or by the NaOH hydrolysis used here, these bands are swamped by the contribution of acetyl groups which known to be located on a fraction of the glucomannans. Since acetyl groups have some freedom to rotate and are not load-bearing, their absorption bands are expected to show relatively little perturbation under mechanical stress. It is quite difficult to find distinctive FTIR bands associated with load-bearing features of xylans *in muro*, a band at 946 cm^-1^ being most promising^7^. The glucomannan band at 810 cm^-1^, transverse to the chain orientation, originates from a non-loadbearing structural feature and is therefore more useful in polarisation studies of reorientation^19^ than in bandshift studies, where the 1085 cm^-1^ glucomannan band is more appropriate.

The C-O-C stretching band around 1160-1165 cm^-1^, which shows strong bandshift and polarisation responses under load, has often been considered to be characteristic of cellulose. This is not the case: it contains contributions from similar structural features in all the polysaccharides in the wood cell wall and from lignin. It is probably dominated by the contributions of different forms of cellulose but these are difficult to distinguish.


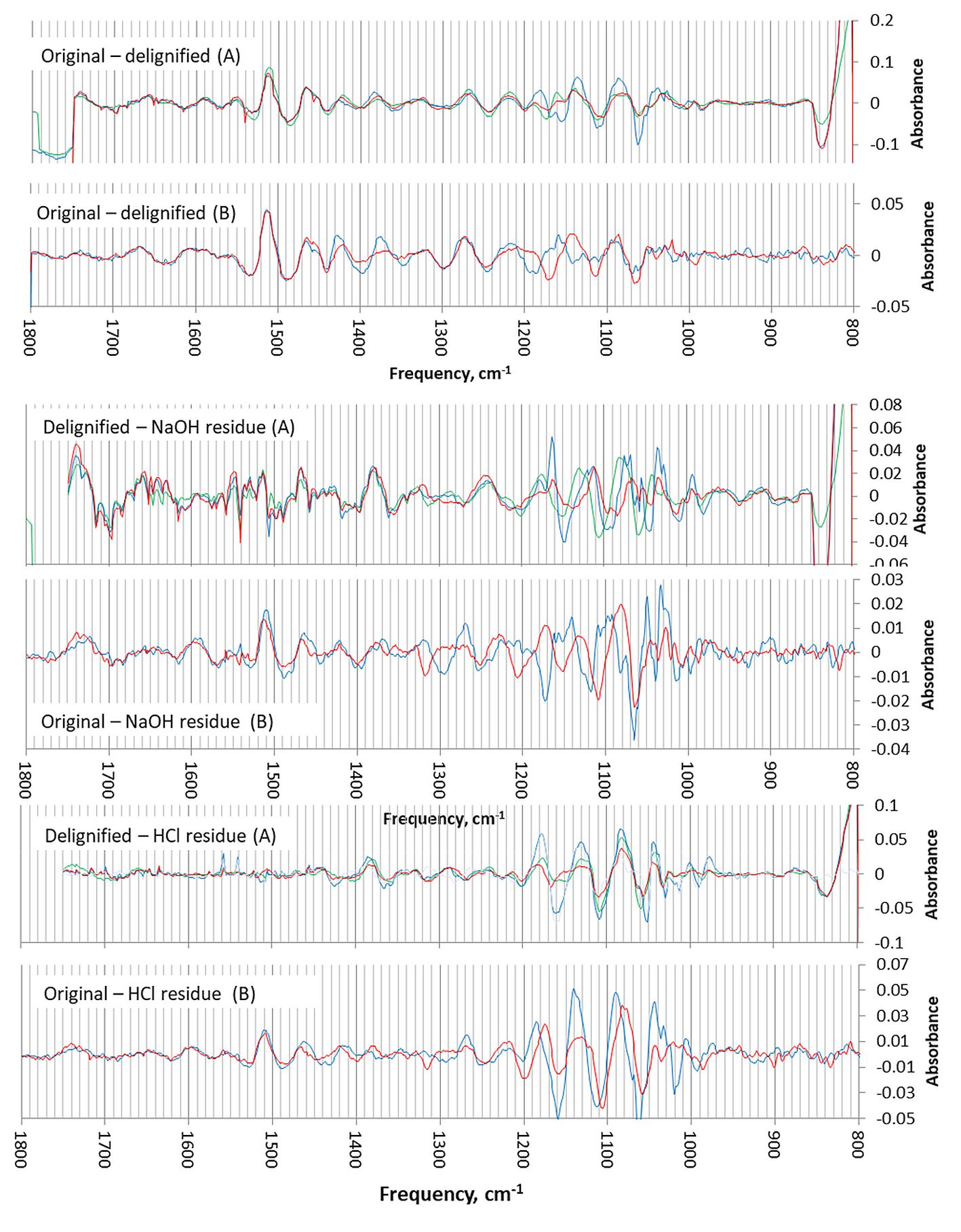


**Fig. S10.** Longitudinally (L) and transversely (T) polarised mean difference spectra used in FTIR band assignment, from sequential extraction including chlorite delignification, extracted with 1M NaOH and hydrolysis with 1M HCl. Two extraction series A and B on different samples.


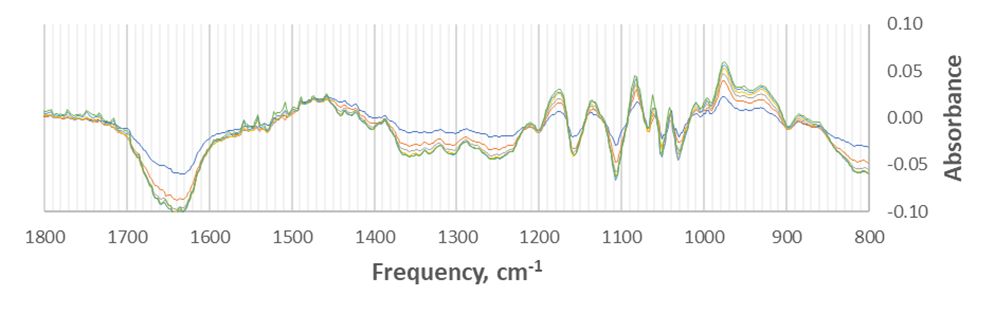


**Fig. S11.** Time-course of (non-polarised) spectral change during deuteration of spruce wood with D_2_O vapour. Difference spectra are from spectra recorded at 5 min (blue), 10 min (orange), 15 min (grey) 20 min (yellow) and 30 min (green) after commencement of deuteration, with the non-deuterated spectrum subtracted.


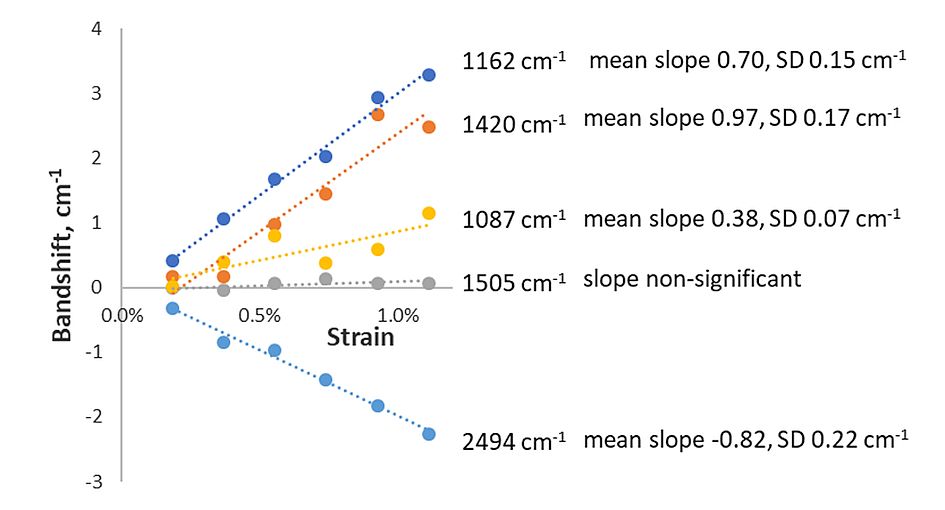


**Fig. S12.** Selected bandshifts in the longitudinally polarised FTIR spectra during progressive tensile elongation of spruce wood internally deuterated under mildly alkaline conditions (*n* = 4). Band assignments: 1162 cm^-1^: predominantly glycosidic C-O-C stretch of cellulose but contains contributions from all other polymers. 1420 cm^-1^: surface cellulose. 1087 cm^-1^: glucomannan. 1505 cm^-1^: lignin. 2494 cm^-1^: O-D stretch from cellulose underlying bound xylan.

**
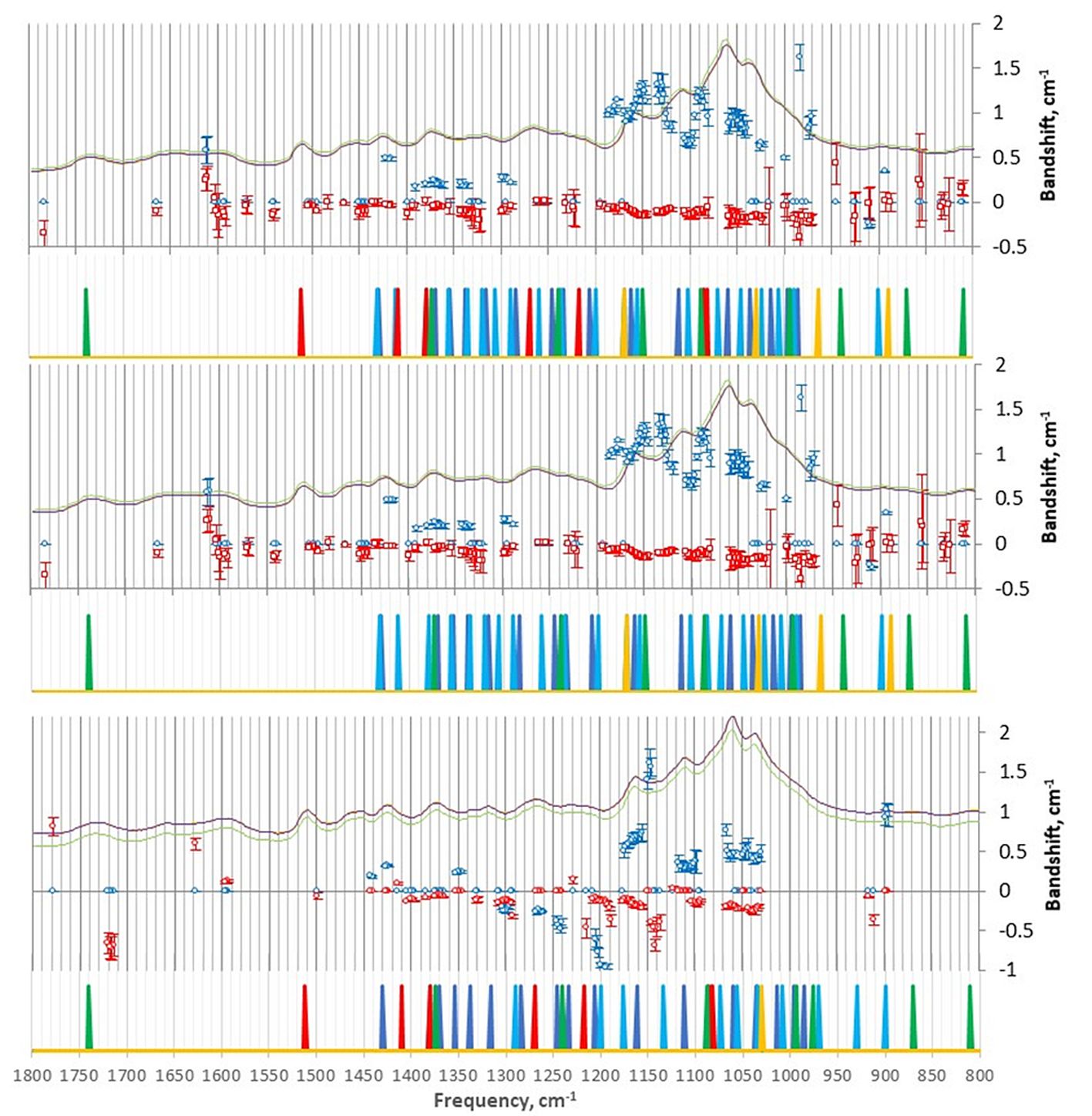
**

**Fig. S13.** Absorbance FTIR spectra and bandshifts for spruce wood during stress-relaxation experiments, in cm^-1^ per 1% tensile strain. A: untreated spruce wood, H form. B: delignified spruce wood, H form. C: untreated spruce wood, vapour deuterated. Error bars indicate 1 SD: *n* = 4 (A and C, *n*=3 (B). Green line: spectra at start, with zero stress. Orange line: spectra after stretching. Violet line: spectra at end of relaxation period. Blue circles: significant bandshifts after initial stress. Red circles: significant bandshifts after 3000 s stress relaxation. Band assignments in bar charts with colour key: dark blue, interior cellulose; light blue, surface cellulose; red, lignin; green, glucomannan including acetyl substituents; orange, xylan. Note that the band assignments differ between the H and D forms: for details see SI.


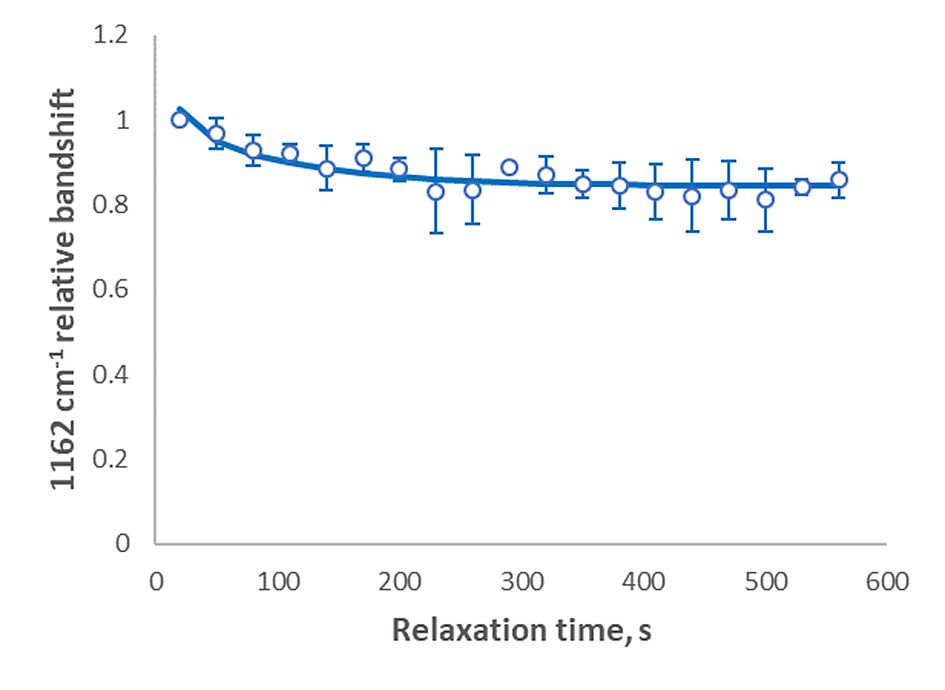


**Fig. S14.** Shift in the relative position of the 1162 cm^-1^ FTIR peak during the relaxation of spruce wood. The 1162 cm^-1^ peak is assigned predominantly to the glycosidic C-O-C stretch of cellulose but contains contributions from all other polymers. The fitted curve is a biexponential function based on the two time constants, 0.84 s^-1^ and 0.17 s^-1^, measured on spruce sections of the same 20 μm nominal thickness (Table S1). Error bars indicate 1 SD (*n* = 3).


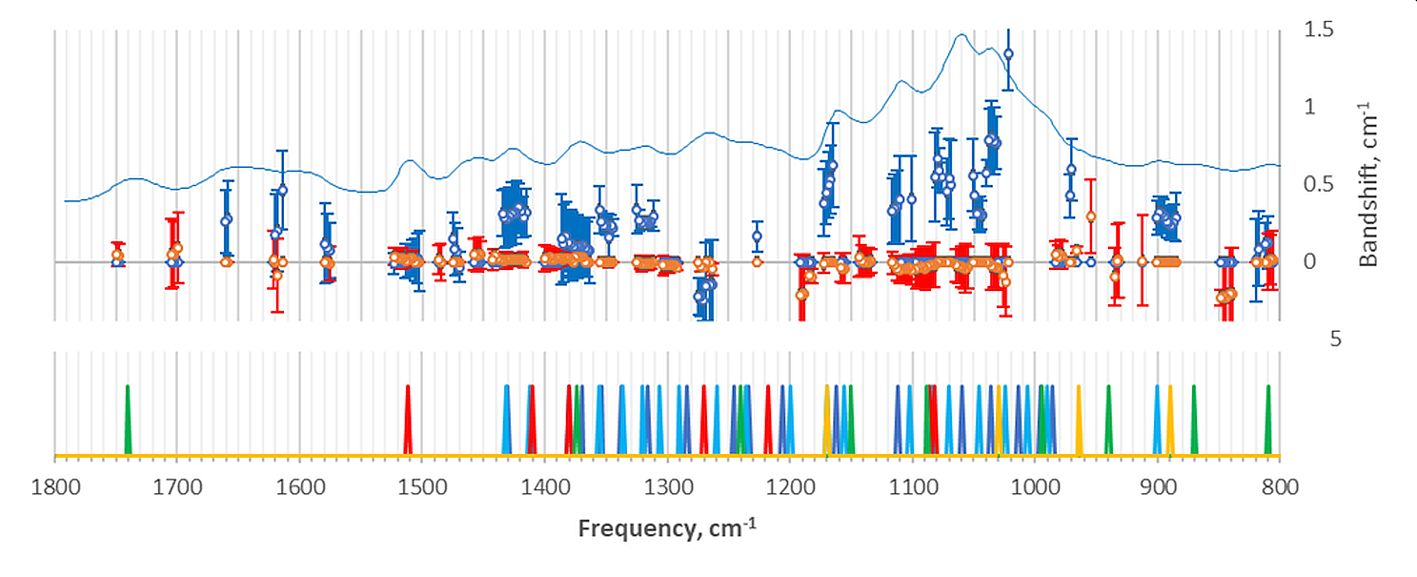


**Fig. S15.** Absorbance FTIR spectra and bandshifts for spruce wood during creep experiments. Blue line: spectra at start. Blue circles: significant bandshifts (cm^-1^) after initial stress. Red circles: significant bandshifts (cm^-1^) after creep at constant load for 30 min. Bar chart: color-coded assignments as in Fig. 6. Error bars indicate 1 SD (*n* = 5).

Supplementary References

1 Guo, F., Altaner, C. & Jarvis, M. Thickness-dependent stiffness of wood: potential mechanisms and implications. *Holzforschung* **In press** (2020).

2 Lichtenegger, H. C., Muller, M., Wimmer, R. & Fratzl, P. Microfibril angles inside and outside crossfields of Norway spruce tracheids. *Holzforschung* **57**, 13-20 (2003).

3 Altaner, C. M., Thomas, L. H., Fernandes, A. N. & Jarvis, M. C. How cellulose stretches: synergism between covalent and hydrogen Bonding. *Biomacromolecules* **15**, 791-798, doi:10.1021/bm401616n (2014).

4 Thomas, L. H., Martel, A., Grillo, I. & Jarvis, M. C. Hemicellulose binding and the spacing of cellulose microfibrils in spruce wood. *Cellulose* **27**, 4249-4254, doi:10.1007/s10570-020-03091-z. (2020).

5 Thomas, L. H., Altaner, C. M. & Jarvis, M. C. Identifying multiple forms of lateral disorder in cellulose fibres. *Journal of Applied Crystallography* **46**, 972-979, doi:10.1107/s002188981301056x (2013).

6 Wise, L. E., Murphy, M. & d'Addieco, A. A. Chlorite holocellulose, its fractionnation and bearing on summative wood analysis and on studies on the hemicelluloses. *Paper Trade Journal* **122**, 35-43 (1946).

7 Chen, Z., Hu, T. Q., Jang, H. F. & Grant, E. Multivariate analysis of hemicelluloses in bleached kraft pulp using infrared spectroscopy. *Applied Spectroscopy* **70**, 1981-1993, doi:10.1177/0003702816675363 (2016).

8 Kulasinski, K., Salmen, L., Derome, D. & Carmeliet, J. Moisture adsorption of glucomannan and xylan hemicelluloses. *Cellulose* **23**, 1629-1637, doi:10.1007/s10570-016-0944-8 (2016).

9 Popescu, C.-M. *et al.* Vibrational spectroscopy and X-ray diffraction methods to establish the differences between hardwood and softwood. *Carbohydrate Polymers* **77**, 851-857, doi:10.1016/j.carbpol.2009.03.011 (2009).

10 Penttila, P. A. *et al.* Xylan as limiting factor in enzymatic hydrolysis of nanocellulose. *Bioresource Technology* **129**, 135-141, doi:10.1016/j.biortech.2012.11.017 (2013).

11 Grantham, N. J. *et al.* An even pattern of xylan substitution is critical for interaction with cellulose in plant cell walls. *Nature Plants* **3**, 859-865, doi:10.1038/s41477-017-0030-8 (2017).

12 Driemeier, C., Mendes, F. M. & Ling, L. Y. Hydrated fractions of cellulosics probed by infrared spectroscopy coupled with dynamics of deuterium exchange. *Carbohydrate Polymers* **127**, 152-159, doi:10.1016/j.carbpol.2015.03.068 (2015).

13 Lindh, E. L. & Salmen, L. Surface accessibility of cellulose fibrils studied by hydrogen-deuterium exchange with water. *Cellulose* **24**, 21-33, doi:10.1007/s10570-016-1122-8 (2017).

14 Khoshtariya, D. E., Hansen, E., Leecharoen, R. & Walker, G. C. Probing protein hydration by the difference O-H (O-D) vibrational spectroscopy: Interfacial percolation network involving highly polarizable water-water hydrogen bonds. *Journal of Molecular Liquids* **105**, 13-36, doi:10.1016/s0167-7322(03)00009-6 (2003).

15 Šturcová, A., His, I., Apperley, D. C., Sugiyama, J. & Jarvis, M. C. Structural details of crystalline cellulose from higher plants. *Biomacromolecules* **5**, 1333-1339, doi:10.1021/bm034517p (2004).

16 Chang, S.-S., Salmén, L., Olsson, A.-M. & Clair, B. Deposition and organisation of cell wall polymers during maturation of poplar tension wood by FTIR microspectroscopy. *Planta* **239**, 243-254, doi:10.1007/s00425-013-1980-3 (2014).

17 Akerholm, M. & Salmén, L. Dynamic FTIR spectroscopy for carbohydrate analysis of wood pulps. *J. Pulp Pap. Sci.* **28**, 245-249 (2002).

18 Akerholm, M. & Salmén, L. Softening of wood polymers induced by moisture studied by dynamic FTIR spectroscopy. *J. Appl. Polym. Sci.* **94**, 2032-2040 (2004).

19 Akerholm, M. & Salmén, L. Interactions between wood polymers studied by dynamic FT-IR spectroscopy. *Polymer* **42**, 963-969 (2001).
